# Advanced Methods for Natural Products Discovery: Bioactivity Screening, Dereplication, Metabolomics Profiling, Genomic Sequencing, Databases and Informatic Tools, and Structure Elucidation

**DOI:** 10.3390/md21050308

**Published:** 2023-05-19

**Authors:** Susana P. Gaudêncio, Engin Bayram, Lada Lukić Bilela, Mercedes Cueto, Ana R. Díaz-Marrero, Berat Z. Haznedaroglu, Carlos Jimenez, Manolis Mandalakis, Florbela Pereira, Fernando Reyes, Deniz Tasdemir

**Affiliations:** 1 Associate Laboratory i4HB—Institute for Health and Bioeconomy, NOVA School of Science and Technology, NOVA University Lisbon, 2819-516 Caparica, Portugal; 2 UCIBIO—Applied Molecular Biosciences Unit, Chemistry Department, NOVA School of Science and Technology, NOVA University of Lisbon, 2819-516 Caparica, Portugal; 3 Institute of Environmental Sciences, Room HKC-202, Hisar Campus, Bogazici University, Bebek, Istanbul 34342, Turkey; enginbayram@reotek.com.tr (E.B.); berat.haznedaroglu@boun.edu.tr (B.Z.H.); 4 Department of Biology, Faculty of Science, University of Sarajevo, 71000 Sarajevo, Bosnia and Herzegovina; llbilela@pmf.unsa.ba; 5 Instituto de Productos Naturales y Agrobiología—CSIC, 38206 La Laguna, Spain; mcueto@ipna.csic.es (M.C.);; 6 Instituto Universitario de Bio-Orgánica (IUBO), Universidad de La Laguna, 38206 La Laguna, Spain; 7 CICA- Centro Interdisciplinar de Química e Bioloxía, Departamento de Química, Facultade de Ciencias, Universidade da Coruña, 15071 A Coruña, Spain; carlos.jimenez@udc.es; 8 Institute of Marine Biology, Biotechnology and Aquaculture, Hellenic Centre for Marine Research, HCMR Thalassocosmos, 71500 Gournes, Crete, Greece; mandalakis@hcmr.gr; 9 LAQV, REQUIMTE, Chemistry Department, NOVA School of Science and Technology, NOVA University of Lisbon, 2819-516 Caparica, Portugal; florbela.pereira@fct.unl.pt; 10 Fundación MEDINA, Avda. del Conocimiento 34, 18016 Armilla, Spain; fernando.reyes@medinaandalucia.es; 11 GEOMAR Centre for Marine Biotechnology (GEOMAR-Biotech), Research Unit Marine Natural Products Chemistry, GEOMAR Helmholtz Centre for Ocean Research Kiel, Am Kiel-Kanal 44, 24106 Kiel, Germany; dtasdemir@geomar.de; 12 Faculty of Mathematics and Natural Science, Kiel University, Christian-Albrechts-Platz 4, 24118 Kiel, Germany

**Keywords:** blue biotechnology, natural products, high-throughput screening (HTS), mode of action (MoA), molecular networking, dereplication, natural products databases, Global Natural Product Social Molecular Networking (GNPS), informatic chemometrics, high throughput next-generation sequencing (HT/NGS), computer assisted structure elucidation (CASE), relative and absolute configuration determination in structure elucidation

## Abstract

Natural Products (NP) are essential for the discovery of novel drugs and products for numerous biotechnological applications. The NP discovery process is expensive and time-consuming, having as major hurdles dereplication (early identification of known compounds) and structure elucidation, particularly the determination of the absolute configuration of metabolites with stereogenic centers. This review comprehensively focuses on recent technological and instrumental advances, highlighting the development of methods that alleviate these obstacles, paving the way for accelerating NP discovery towards biotechnological applications. Herein, we emphasize the most innovative high-throughput tools and methods for advancing bioactivity screening, NP chemical analysis, dereplication, metabolite profiling, metabolomics, genome sequencing and/or genomics approaches, databases, bioinformatics, chemoinformatics, and three-dimensional NP structure elucidation.

## 1. Introduction

**Figure 1:**
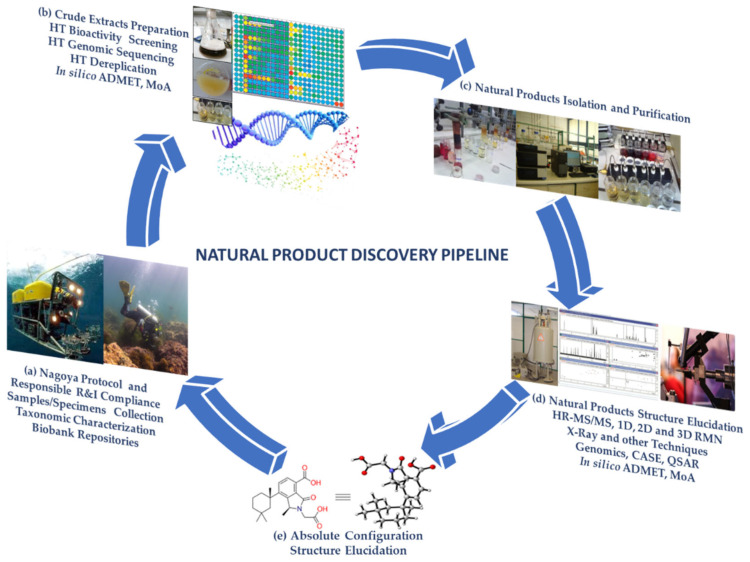
Natural product discovery workflow. (**a**) NP and RRI sampling, taxonomic characterization, biobank repositories; (**b**) HT bioactivity screening, genomic sequencing, dereplication, in silico preclinical trials; (**c**) NP isolation and purification (out of the scope of this review); (**d**) structure elucidation, methods for attaining the NP 3D chemical structure, in silico preclinical trials; and (**e**) NP elucidated with its absolute configuration.

Natural bioresources are well known for producing secondary metabolites with unique features, highly complex structures, and biochemical properties valuable for human healthcare and well-being, which have inspired industries for numerous biotechnological applications [1,2]. The urge to fill the industrial pipelines and to identify novel lead-like compounds for drug discovery that can meet the challenge of lacking suitable therapeutic agents for a wide range of diseases is very high [3]. This comprehensive review covers the high-throughput (HT) workflow for natural product (NP) discovery, from bioassay screening, docking, and mode of action (MoA) prediction to HT analytical equipment, metabolomics, genomics, NP databases, in silico computational approaches that support NP dereplication (early identification of known compounds), metabolite profiling, quantitative structure activity relationship (QSAR), and computer assisted structure elucidation (CASE), as well as methods for the determination of secondary metabolites relative and absolute configuration to elucidate their 3D chemical structure, with particular focus on methodological prospects and advances (Figure 1).

Two major bottlenecks that hinder NP discovery are dereplication and structure elucidation, particularly the determination of the relative and absolute configuration of secondary metabolites with stereogenic centers. Herein, particular focus will be given to these subjects. Dereplication has become a hot topic in the past decade, with nearly 1240 publications (Web of Science) and 908 articles published after April 2014 that have received over 40,520 citations in total. In the pursuit of Marine Natural Products (MNP), Blue Biotechnology (BB), which is the application of science and technology to living aquatic organisms to produce knowledge, goods, and services (OECD, 2016), brings together multiactors and multidisciplinary fields, blending them in new ways such as combining organic and analytical chemistry with molecular biology, genomics, and/or informatic chemometrics, thus providing key conceptual or methodological advances that are likely to open innovative research possibilities. Some of the NP methods are so intricately connected that it is very difficult to separate them into sections without overlapping. Our insights into this theme will give priority to studies that reported significant advances in the field, highlighting the major advances that have shaped the ground, including method comparisons, our perspective on developments, future trends, and the carving of new directions. This review is meant to be complementary to our highly cited 2015 Natural Products Report (NPR) paper, entitled “*Dereplication: racing to speed up the natural products discovery process*” [4].

Since April 2014 to January 2023, eighty-nine reviews have been published out of the 908 published papers on NP dereplication, while 387 papers were reported in the ambit of NP structure elucidation, with 40 of these considering the determination of molecular relative and absolute configuration. These include highly cited and recent reviews covering: (1) integration of taxonomic and/or bioactivity data [5]; (2) the analysis of the chromatographic hyphenated techniques (LC-MS, GC-MS, and LC-NMR) with spectrometric (MS/MS) and spectroscopic data (NMR) for metabolite profiling [6]; (3) a comprehensive overview of NP databases, with emphasis on free open access databases [7,8,9,10,11,12,13]; (4) molecular networking strategies for NP dereplication and distinct dereplication workflows [14,15,16,17,18]; (5) dereplication using metabolomics, genomics, and metagenomics [19,20,21,22,23,24]; and (6) in silico methods (artificial intelligence and machine learning) for dereplication and structure elucidation [25,26]. With regard to computational/bioinformatics tools, the reviews of Medema et al. [27] and Ren et al. [28], both published in 2020, are suggested, while the paper by Prihoda et al. [29] is recommended for machine learning (ML) methods in NP discovery. 

Unambiguous stereochemical assignments of NP remain a challenge. In this context, we highlight reviews on: (1) reassignment of absolute configuration [30,31]; (2) NMR calculation with quantum chemical approaches, such as DP4 [32,33,34,35,36,37], including optical rotation, and electronic and vibrational circular dichroism aided by quantum chemical calculations [35,38]. There are several reviews that address the developments in computer-assisted structure elucidation (CASE) systems [4,39,40,41,42]. Among them, we highlight that 3D structure analysis in conjunction with CASE can be performed not only by including 2D NOESY/ROESY experimental data [39,40,41], but also by using DFT chemical shift analysis [35,40,43,44,45]; and (3) structure elucidation aided by genomics [46,47,48,49,50,51]. Many reviews that systematically list the most used tools of synthetic biology methodologies have been published from mid-2014 to date. For reviews on microbial genome mining, these particularly focus on genome mining strategies and tools for ribosomally synthesized and post-translationally modified peptides (RiPPs) [48,52,53]. The work of Robinson et al. [54] is suggested as an excellent review with a roadmap on metagenomic enzyme identification.

The authors of this review are members of the COST Action CA18238—European Transdisciplinary Networking Platform for Marine Biotechnology (https://www.ocean4biotech.eu/ (accessed on 23 March 2023)) [55,56]. Thus, although the techniques described in this review can be used both for NP of terrestrial and marine origin, we chose, whenever possible, to give examples of MNP.

Discovering unique MNP presents added challenges such as accessing organisms in extreme or deep environments, reviving uncultivable microorganisms under lab conditions, dereplication, solving sustainable supply issues, discovering their bioactivity and mode of action (MoA), and optimizing their pharmacological properties [51,57]. However, efforts made in these directions have been rewarded, as MNP are a promising source of medicines, with 17 marine-derived drugs successfully approved and several other marketable marine-derived products. The development of innovative discovery approaches in the fields of screening methods, metabolomics, genomics, metagenomics, proteomics, combinatorial biosynthesis, synthetic biology, expression systems, and bioinformatics, combined with dereplication, will continue to unravel MNP with unique structural and biological properties and MoA for numerous biotechnological purposes [58,59]. 

It is our goal to give insight to the BB community on the most advanced HT methods for MNP discovery and knowledge on structure elucidation. We believe that this review may be used as a guideline for the whole NP discovery process in academic laboratories.

## 2. Advances, Trends, and Challenges in High-Throughput Screening (HTS)

The following section summarizes a set of selected methods and studies that have attracted great attention from the research community since April 2014 (based on annual citation rate and/or total number of citations). Consideration has been given to HTS studies referring to MNP and approved drugs.

### 2.1. Lab-Based HTS

A recent review restates the decreasing enthusiasm of major pharmaceutical companies for implementing HTS programs, particularly on NP [49]. It was reported that besides legitimate concerns (e.g., regulations on international access to natural bioresources), biological extracts are typically too complex to be compatible with HTS for specific molecular targets, and the costly efforts tο reduce chemical complexity make the whole procedure less attractive. The limited success of large HTS campaigns previously performed by companies was deemed to be another reason for the decreasing interest in the pharmaceutical industry, though the interest in HTS and NP for drug discovery remains a hot research topic in academia. 

Navarro et al., 2014 designed an image-based 384-well HTS method for the discovery of biofilm inhibitors and inducers of biofilm detachment against the biofilm-forming pathogen *Pseudomonas aeruginosa* [60]. This method uses non-z-stack epifluorescence microscopy to image a constitutively expressing green fluorescent protein (GFP)-tagged strain of *P. aeruginosa*, and the quantification was performed using an automated image analysis script. Bacterial cellular metabolic activity in combination with biofilm coverage was measured using the redox-sensitive dye XTT to distinguish between antibiotics and nonantibiotic biofilm inhibitors [60].

Caicedo et al., 2017 developed data-analysis strategies for image-based cell profiling, a high-throughput method for the quantification of phenotypic differences among a variety of cell populations, using image acquisition with high-throughput microscopy systems and subsequent image processing and analysis. This method enables the design of experiments for several biological objectives [61].

Laubscher and Rautenbach, 2022 developed an effective preliminary screening assay to identify antibacterial-producing bacteria called the bioluminescent simultaneous antagonism (BSLA) assay, which measures the luminescence of bioluminescent reported bacteria co-cultivated in 96-well plates with bacterial isolates under investigation to determine the production of antibacterial compounds. The authors argued that this assay is amenable to scaling up and can be incorporated into automated HTS systems, permitting rapid pre-screening of unknown bacterial isolates, which, when coupled with dereplication and identification technologies, can effectively fast-track antimicrobial discovery [62].

In 2022, Orlov et al. designed a workflow that included molecular component analysis with High Resolution Mass Spectrometry (HR-MS), selective chemical tagging and deuterium labeling, liver tissue penetration analysis, in vitro evaluation of biological activity, and computational chemistry tools used to produce putative structural drug-lead candidates. A proteomic experiment was also carried out to evaluate the potential MoA of these suggested structures by molecular docking [63].

Drug repurposing (i.e., the identification of existing medicines with established safety for the treatment of new and rare diseases) is a smart strategy for increasing popularity in HTS campaigns as it reduces the cost, effort, and time required for drug development. This approach is particularly attractive in emergency situations such as COVID-19. Chen et al. employed a SARS-CoV-2 cytopathic assay with an accompanying cytotoxicity counter-assay to screen 8810 approved/investigational drugs, bioactive compounds, and NP at four different concentrations [64]. A total of 319 hits with antiviral activity were found, with almost half of these being approved/investigational drugs. Chlorprothixene, methotrimeprazine, and piperacetazine were the three most potent FDA-approved drugs that were repurposed for the fight against coronavirus. 

### 2.2. Structure-Based Virtual HTS, MoA Prediction, New Trends, and Challenges

Bertrand et al., 2016 investigated the potential of statistical correlation analysis to enable unambiguous identification of features related to bioactive compounds in crude extracts without the need for compound isolation using UHPLC-ESI-TOFMS profiles, micro-flow CapNMR spectra, an anticancer bioassay, and statistical correlation analysis, enabling early-stage detection of the compounds bioactivity [65].

Bioactive Molecular Networking (BMN) was designed in 2018 by Dorrestein and co-workers as a bioinformatic pipeline to find candidate active molecules directly from bioactive extracts, aiming to avoid the isolation of non-bioactive compounds from bioactive extracts. This tool enables mapping bioactivity scores in MN and can speed up the process of drug-lead discovery by revealing bioactive secondary metabolites in complex mixtures without previous compound isolation [66]. MASST is an informatic tool incorporated in the Global Natural Product Social Molecular Networking (GNPS), described in Section 4.2.1, that may feasibly incorporate translation of in vitro or in vivo data from model organisms to humans [67]. 

In a recent study, six marine-derived *Streptomyces aculeolatus* extracts were analyzed by LC-MS/MS, and the data were scrutinized by MN in conjunction with supervised multivariate statistical analysis and partial least squares discriminant analysis (PLS-DA) to unveil the correlation between the metabolite classes and antibiofilm activity. Napyradiomycin SF2415B3 inhibition was confirmed for *S. aureus* biofilm formation [68]. Napyradiomycins were later found to exhibit marine antibiofilm and antifouling activity [69].

Blanco et al., 2020 introduced a pipeline designated EasyDIVER (Easy pre-processing and Dereplication of In Vitro Evolution Reads), which facilitates the computational analysis of HTS data from in vitro evolution experiments and selection trials for the discovery of functional RNA nucleic acids and peptides. This pipeline supports the input of raw, paired-end, demultiplexed raw files, providing dereplicated unique nucleic acid and/or peptide sequences and their count reads [70]. 

GraphAMR, a novel computational workflow available at https://github.com/ablab/graphamr (accessed on 23 March 2023), enables the recovery and identification of antibiotic resistance genes from fragmented metagenomic assemblies [71]. The availability of extensive (meta)genomic datasets has started complementing bioactivity-guided screening of bacterial extracts and the characterization of biosynthetic pathways for drug discovery, ushering researchers into the post-genomics, big-data era [50].

Understanding the MoA of complex mixtures early in the NP discovery pipeline is important to define their practical applications. In 2015, Linington and co-workers developed a new platform, entitled Compound Activity Mapping (CAM), which directly predicts the identities and MoA of bioactive constituents of complex NP extract libraries. This new tool identified novel bioactive compounds and predicted the compounds MoA based on primary screening data. In essence, it converted the NP discovery workflow into a targeted, hypothesis-driven discovery model where the chemical properties and biological MoA of the bioactive metabolites are known early in the screening process and the lead NP can be rationally selected based on biological and/or chemical novelty [72]. Recently, this methodology evolved into an open online CAM platform, termed NP Analyst, available at www.npanalyst.org (accessed on 23 March 2023), which integrates biological screening and untargeted mass spectrometry (MS) library data for NP discovery, complementing current discovery workflows. NP Analyst is compatible with almost any type of bioassay data, MS data via the mzML format, as well as processed MS data from MZmine and GNPS open-source platforms [73]. Another recent study performed by O’Rourke et al. established a MoA classification method using global transcriptome profiling [74].

In the same context, the cytotoxic activity was examined in the crude extract and respective fractions derived from the Red Sea sponge *Amphimedon* sp. The chemical constituents identified in the active fraction by LC-MS analysis were subjected to molecular docking against the active site of SET oncoprotein. Amphiceramides A-B, as well as acetamido glucosyl ceramide revealed the highest energy binding affinities and interactions with the binding site of this protein. Additionally, ADME/Tox calculations were performed for these MNP to predict their pharmacokinetic profile [75]. 

We further distinguished a few recent studies dealing with the challenges/limitations or providing some new trends in HTS. Following the success in biomedical research, zebrafish (living embryos of *Danio rerio*) is gaining a growing interest as a model for high-content HTS (i.e., automated, image-based morphological profiling of biological activity in cells or whole organisms) in drug discovery programs. Besides investigating the therapeutic effect of a molecule, zebrafish embryos can facilitate other steps of the discovery process, including target validation, toxicity evaluation, and drug optimization. In the study of Gallardo et al. [76], a total of 2960 chemicals, including 800 NP, were screened in zebrafish embryos, and 165 compounds inhibiting primordium migration without overt toxicity were identified as potential antimetastatic agents. The ability of the inhibitor SU6656 to decrease tumor metastasis was subsequently confirmed with in vivo experiments in a mouse tumor model.

Regarding strategic actions promoting HTS-driven drug discovery, it is worth mentioning the initiative led by the US National Cancer Institute [77]. To stimulate HTS efforts and accelerate NP drug discovery, the NCI Program for Natural Product Discovery (NPNPD) was launched in 2018 to create a publicly accessible HTS-amenable library of over 1,000,000 fractions from 125,000 marine, microbial, and plant extracts collected from around the world. About 326,000 fractions were made available in 384-well plates, free of charge and open to screening against any disease target by 2019 (https://dtp.cancer.gov (accessed on 23 March 2023)). 

There is no doubt that HTS continues to be a key strategy for identifying chemical compounds capable of inhibiting or activating specific disease-related targets, while new assays are constantly being developed to support drug discovery efforts. Though the discussion about the common artifacts in HTS-derived hits has raged for the last 5 years, it has also highlighted the importance of avoiding particularly high concentrations during cell-based screening of NP against specific biological processes [78]. This debate was particularly focused on molecules presenting a strong effect in a wide variety of assays, which are commonly referred to as pan-assay interference compounds (i.e., PAINS) [79,80]. Demonstrating non-specific binding/interaction with proteinaceous targets, PAINS are frequently identified as positive hits in HTS programs and incorrectly assumed to possess drug-like properties. Such confusing situations are encountered in the screening of both synthetic drugs and NP [79,81]. There is a growing consensus that hits with promiscuous activity profiles (e.g., isothiazolones, toxoflavin-like, quinones, etc.) should be excluded from further investigation when drug discovery projects are focused on the one-drug-one-target paradigm using biochemical assays (molecular target-based) [80,82], but some researchers advocate that this practice can be detrimental when implementing cell-based phenotypic screening [82]. Despite the conflicting viewpoints on this issue, scientists dealing with HTS should be more vigilant and cautious about PAINS-induced artifacts to avoid wasting time and effort on worthless experiments.

## 3. Advances in HT Analytical Techniques for NP Dereplication

The high separation efficiency and the enhanced capability for hyphenation with a wide variety of detection systems such as UV-VIS/DAD, ELSD, MS, HR-MS, HR-MS/MS, and NMR make High Performance Liquid Chromatography (HPLC) or Ultra High Performance Liquid Chromatography (UHPLC) (when using columns packed with sub−2 µm that require higher pressure levels) the most common separation techniques used in the early stages of NP dereplication studies [9,83]. 

Due to its extreme sensitivity, rapidity, and ability to identify even very complex mixtures, liquid chromatography coupled with mass spectrometry (LC-MS) is nowadays the most widely used method for untargeted metabolomics and dereplication of MNP. 

Overall, the annotation rate of LC-MS-based untargeted metabolomics is around 2–5%. Hence, most of the chemical signatures of a biological organism remain unannotated [84,85]. The need for more effective annotation of metabolites led to the development of mass spectrometry instruments with higher resolution for dereplication. An added benefit of LC-HR-MS systems is their capability to analyze numerous samples in a short time, using minimal quantities of biological extracts, and attaining an increasingly growing amount of analytical data. Despite these advantages, HR-MS is unable to distinguish and identify co-eluting isomeric and isobaric compounds [86], but increasing progress has been observed in this direction with the recent advent of systems integrating ion mobility separation.

In detail, classical MS1-type full-scan metabolomics often gives limited information regarding the novelty of the compounds (i.e., presence/absence in a database or databases), and they do not provide insights about the existence of structural analogs or derivatives, hence limiting the value of the collected data in terms of chemical annotation [87]. Another major challenge faced by the MS1 approach is that many structurally unrelated compounds share the same molecular formula and mass [88], and hence they cannot be distinguished using mass spectrometric data alone [86]. In contrast, HR-MS/MS (MS2) spectra are specific to chemical families, and nowadays this hyphenated technique has become the most preferred method for MNP dereplication studies. This is because the chemical structure of a compound determines how it will be fragmented by MS/MS in the gas phase; thus, molecules that share the same core structure will exhibit very similar fragmentation patterns [87].

The mass spectrometers equipped with collision cells that are capable of producing MS2 ions from molecular ions using different fragmentation mechanisms [(e.g., Collision Induced Dissociation (CID), Higher Energy Collisional Dissociation (HCD), Electron-Transfer Dissociation (ETD), Electron Activated Dissociation (EAD), etc.] and the hybrid systems combining different types of mass analyzers (i.e., Q-TOF, LTQ-Orbitrap) have remarkably increased the informative power of the MS detectors (especially for HR-MS/MS) [86,89,90]. Orbitrap equipment is among the most commonly used hyphenated analytical instruments for dereplication purposes, as GNPS only accepts Data-Dependent Acquisition (DDA) data, i.e., molecules fragmented with CID, HCD, or ETD, and only supports for analysis the file formats .mzXML, .mzML, and .mgf (https://ccms-ucsd.github.io/GNPSDocumentation/isgnpsright/ (accessed on 23 March 2023)).

NP isolation and purification are beyond the scope of this review. Nevertheless, chromatographic techniques are the most commonly used for this purpose, either using normal or reverse-phase silica, depending on the NP polarity, alumina for NP that require neutral pH conditions, or Sephadex for molecular weight-based isolation. It is also very common to perform pre-fractionations by column chromatography, followed by semipreparative or preparative HPLC chromatography.

## 4. Dereplication Advances, Databases, Informatic Tools, and Case Studies

The rapid identification of previously reported compounds, termed as structural dereplication, is a crucial component in NP and MNP chemistry. The taxonomic characterization of the metabolite-producing organisms, the availability of molecular structure data for known metabolites, and the accessibility to metabolite spectrometric and spectroscopic signatures are considered the focal points of structural dereplication [91]. 

Enabling free, open access to databases will advance new technologies in NP discovery. Increased progress on new computational methodologies for secondary metabolite identification and elucidation will be achieved by enhancing and improving comprehensive databases of known compounds to compare against experimental data [92]. In addition, further advances in the creation of hybrid platforms that combine the advantages of hyphenated chromatographic techniques (LC-MS, GC-MS, and LC-NMR), especially those involving HR-MS/MS detection, computational MS, and MS/MS prediction methods, are needed to enhance the power of metabolomics and enable more efficient, accurate annotation and dereplication in NP research. Additionally, the synergy created by combining these techniques enables nearly unlimited access to the NP chemical space [93]. 

### 4.1. LC-MS/MS Data Visualization and Annotation Methods

Comparing untargeted metabolomics data produced by several laboratories is difficult, but the application of Principal Component Analysis (PCA) in data sets with low feature overlap can yield the same qualitative description of a sample set [94]. Simplified PCA models using Planes of Principal Component Analysis in R (Pearson coefficient, R) (PoPCAR) identify *m*/*z* or molecules that are exclusive to each strain within a group, supporting automated mass matching to databases such as Antibase [95].

Molecular networking (MN) and substructure-based MN (MS2LDA), which identify shared structural motifs [96], were developed as molecular mining tools for the discovery of molecular families and substructures in MS/MS data. This approach enables the perception of small molecular changes within samples, advancing research as a result of the refined organization of MS/MS data [85,97]. 

MN was originally introduced in 2012 [97]. It connects molecules based on their fragment ion mass spectra (MS/MS) and uses a vector-based computational algorithm to mine/compare the spectral similarity of MS/MS spectra in large datasets. The output is visualized by software as networks of MS/MS spectra, i.e., molecular networks, where the nodes represent each molecule and the thickness of the edges connecting the nodes indicates the structural similarity of NP sharing the same biochemical origin [87]. MN *per se* does not allow searching for NP, but it found enormous use after the publication of Wang et al. in 2016 [98], being empowered by the GNPS (http://gnps.ucsd.edu (accessed on 23 March 2023)) (Section 4.2.1), a public web-based platform that compiles large volumes of crowdsourced metabolomics datasets [98].

Due to their versatile nature, MN-based approaches combined with GNPS have become an efficient and popular dereplication strategy, representing a breakthrough in the exploration of MS/MS-based untargeted metabolomics of small molecules. 

As a downside, MN may leave adduct species from the same molecular family separated and unconnected. To overcome this issue, Schmid et al., 2021 fused MS- and MS/MS-based networks and integrated them into the GNPS environment, naming this new approach Ion Identity Molecular Networking (IIMN). This approach improved network connectivity for structurally related molecules by integrating chromatographic peak shape correlation analysis into molecular networks to connect and collapse different ion species of the same molecule [99].

In contrast with manual examination of MS/MS spectra connected in the spectral networks, which is only possible when a reference library spectrum is available, in silico predictions emerged as alternative methods to annotate an unknown fragmentation mass spectrum. Nevertheless, the uncertainty around the correct structure among the predicted candidate lists is a disadvantage. The Network Annotation Propagation (NAP) tool available in the GNPS platform, https://gnps.ucsd.edu/ProteoSAFe/static/gnps-theoretical.jp (accessed on 23 March 2023), was developed to improve the accuracy of in silico predictions by generating a network consensus of re-ranked structural candidates using the MN topology and structural similarity and propagating structural annotations even when there is no match to a MS/MS spectrum in spectral libraries [100]. However, LC-MS/MS methods coupled with GNPS have often been overinterpreted, showing results that include absolute configurations.

The major drawback of MN is the low coverage and accuracy of compound annotation due to the limited size of the available databases, as well as the problems in the differentiation of similar chemical scaffolds. Liu et al. 2020 reported an improved MN-based approach, termed Diagnostic Fragmentation-Assisted Molecular Networking coupled with in silico dereplication (DFMN-ISD), to overcome the mentioned obstacles. By adopting rule-based fragmentation patterns, insights into similar chemical scaffolds were provided, while the generation of in silico candidates based on metabolic reactions expanded the coverage of available NP databases, and the in silico annotation methods further facilitated the dereplication of candidates by computing their fragmentation trees [101]. 

Feature-Based Molecular Networking (FBMN) is an analysis method in the GNPS infrastructure that recognizes isomers, incorporates relative quantification, and integrates ion mobility data [102]. By evaluating the effect of data acquisition parameters on the network topology resulting from the Classical Molecular Networking workflow (CLMN) and the new FBMN, it was shown that sample concentration, run duration, collision energy, and the number of precursors per cycle had the greatest influence. While all four parameters were important to optimize for FBMN, the optimization of sample concentration and LC duration was only of high importance for CLMN [103]. Additional methods have been developed for MS/MS-based MN, including the ones mentioned above: Ion Identity MN (IIMN), Building Blocks-Based Molecular Networking (BBMN), and Bioactivity-based MN (BMN) [104]. 

The combination of MN with in silico MS/MS fragmentation tools is also an effective approach for early identification of NP and annotation of their analogues using database entries [105]. Moreover, MN-based approaches coupled with in silico tools can be used to dereplicate Peptidic Natural Products (PNPs), antibiotic metabolites with astonishing diversity, from untargeted MS data acquired on crude extracts to propagate annotations to structurally related molecules [106].

MolNetEnhancer merges multiple independent in silico methods, providing an upgrade in MN through the combination of metabolome mining and annotation approaches. In detail, this workflow incorporates the outputs from MN, MS2LDA, and MS2LDA-MOTIF in silico annotation methods (e.g., NAP or DEREPLICATOR), and the automated classification of chemical entities by ClassyFire, contributing to the identification of unannotated ions [85,107]. Moreover, the SIMILE (Significant Interrelation of MS/MS Ions via Laplacian Embedding) algorithm can interrelate small molecules according to their aligned fragmentation spectra and infer structural connections in MN. In contrast to other alignment methods, this tool calculates the statistical significance of spectral alignment, whereas it is applicable to compounds that have multiple structural differences and produce fragmented ions that are difficult to align [108].

The metabolomics research software MSDIAL and XCMS Online (for processing and annotation of LC-MS/MS data), MetaboAnalyst (for metabolic pathway enrichment and topology analysis), and HMDB (for metabolite identification via MS/MS spectral search), as well as several algorithms developed for MS data analysis, including MN and fragmentation trees, enable similarity searches against known molecules reference libraries or finding statistical relationships between molecular features. However, none of these tools can search a mass spectra against publicly available repositories to track down related or identical MS/MS spectra, including those from unidentified molecules [109].

In addition, Dorrestein and co-workers developed a MN tool for the identification of metal-binding compounds in complex mixtures. After analyzing a sample in a LC-MS/MS system with and without post-column metal infusion, the resulting data are subjected to a comparative analysis using GNPS to identify ion species with the same chromatographic profiles having defined metal-specific mass (*m*/*z*) offsets [110].

The Qemistree workflow (freely available via QIIME2 and GNPS) creates a hierarchical organization of molecular fingerprints predicted from fragmentation spectra and unveils molecular structural relationships among molecules through tree-based representations, providing further support to the annotation process and offering additional confidence in individual identifications [111]. While MN clusters and visualizes closely related metabolites in molecular families, Qemistree calculates all pairwise chemical relationships between different samples using fragmentation trees and supervised machine learning from CSI:FingerID and visualizes them in the context of sample metadata [111]. 

MN combined with whole genome sequencing of intra-species bacterial strains proved to be a successful dereplication strategy [112,113]. The open access tool PPNet, available at (https://github.com/liyangjie/PPNet (accessed on 23 March 2023)), constructs functional association networks of bacterial species from genome-scale data. Through the analysis of phylogenetic profiles with binary similarity and distance measures, it derives large-scale bacterial gene association networks of a single species, allowing a better understanding of pathogenic mechanisms or other biological phenomena of bacteria [114]. Moreover, Chemical Proportionality (ChemProp) scores the changes of abundance between two connected nodes over sequential data series (e.g., temporal or spatial relationships), which allows to prioritize potential biological and chemical transformations or proportional differences of biosynthetically related compounds [115], and EMPress enables visualizing phylogenetic trees in the context of microbiome, metabolome, and other community data [116]. This tool provides some unique functionalities, such as ordination plots of microbiota and animations, together with many standard tree visualization features, making exploratory analyses of various types of omics data easier. 

Optimus and ‘ili software enabled 3D molecular cartography using MS/MS data and following an optimized/standardized methodology. This approach allows for mapping the spatial distribution of small molecules on several environmental and biological surfaces, including the human body, and it is expected to advance various applications in medicine, ecology, agriculture, biotechnology, and forensics [117].

#### 4.1.1. LC-MS/MS Data Visualization and Annotation—Case Studies

In MNP chemistry, MN has been successfully applied to both macro- and micro-organisms to streamline the discovery of new, bioactive metabolites and address diverse research questions. MN-guided exploration of large culture collections allows for rapid dereplication of known molecules and can highlight producers of unique metabolites. These approaches, combined with large culture collections and growing databases, enhance data-driven strain prioritization with a focus on novel chemical scaffolds [118].

One of the earlier applications, performed in 2017 by Crüsemann et al., MN, was applied to a large collection of marine actinobacteria extracts, using marine obligate *Salinispora* and marine-derived *Streptomyces* strains, to explore the effect of different extraction and culture conditions on their chemical profile, thereby prioritizing the most promising ones for further studies [119]. MS/MS analysis and subsequent MN dereplication identified 15 molecular families of diverse MNP and their analogues, allowing to rapidly identify patterns in metabolite production that can be linked to taxonomy, culture conditions, and extraction methods [119]. Fan et al. mapped the One Strain Multiple Compounds (OSMAC)-based culture conditions (different culture regimes and culture media) as well as the anticancer activity and cytotoxicity of marine fungal extracts associated with the brown macroalga *Fucus vesiculosus* onto molecular networks [120]. Bracegirdle et al. [121] profiled the marine tunicate *Synoicum kuranui* by MN and showed the presence of two new methylated rubrolides (non-nitrogenous polyaromatic butenolides). Both compounds were isolated by MS-guided fractionation and showed strong antimicrobial activity. In another study guided by MN-based metabolomics and cytotoxic activity [122], two new oligomeric pyrroloiminoquinone alkaloids were isolated. These corresponded to tridiscorhabdin, the very first trimeric discorhabdin molecule reported from Nature, and the dimeric didiscorhabdin, both of which contained a novel C-N bridge between discorhabdin monomers. The use of an additional statistical method (Pearson coefficient, R) allowed the prediction of bioactivity scores of molecules in molecular networks, and this approach has been applied to marine fungi, yeast, and seaweeds [120,123,124]. Another example of the use of MN and GNPS was performed by Bauermeister et al. for the identification of variances in secondary metabolite production by *Salinispora pacifica* and *Salinispora arenicola* species isolated from different locations, specifically islands situated in the North and South Atlantic Oceans [125]. 

Combining MN with pattern-based genome mining in 35 *Salinispora* species, the quinomycin-type depsipeptide retimycin A was discovered and structurally characterized. The biosynthesis of this compound was linked to the gene cluster NRPS40 using pattern-based bioinformatic approaches [126]. 

One example of the use of MS/MS Qemistree representation performed by Pinto-Almeida et al. revealed similarities in fatty acids among marine-derived *Micromonospora* and *Streptomyces* strains and macrolactams and prenol lipids among *Streptomyces* strains [127].

### 4.2. Dereplication Using LC-MS/MS, NP Databases, and Informatic Tools

NP databases play an essential role in structural MS-based dereplication efforts. Constant improvements made over the last few years in analytical tools and their availability in most laboratories have been paralleled with the development of commercial and free open access databases to assist NP chemists in their efforts to identify known compounds present in natural extracts. Herein, we will highlight the most recent developments dedicated to MS dereplication, as well as general-purpose structural databases, and their contribution to the NP discovery global effort. 

#### 4.2.1. GNPS Database, GNPS-Combined Databases, Integrated Analytical and Informatic Tools, and Other NP Databases to Aid LC-MS/MS Dereplication

The GNPS database/platform comprise the most powerful informatics tools in NP dereplication [98]. This is an online open access small molecule tandem mass spectrometry (MS/MS) data community-curated and analysis platform for untargeted metabolomics without the need for isotopic labeling. As previously mentioned, it is available at (http://gnps.ucsd.edu (accessed on 23 March 2023)). It completely shaped the way of performing dereplication using data-driven social networking of molecules, facilitating spectra identification, high-throughput annotation of NP in mixtures, finding novel analogues in desired structural classes, identifying new chemical entities, and promoting worldwide collaborations. Compared to previous NP databases, which were non-searchable with raw MS/MS data and did not allow community sharing of raw spectra, this infrastructure made a great step forward, and it is now the most utilized among the NP research community [98]. MS/MS molecular networking analysis integrated with GNPS annotation is compatible with high-throughput extract analysis, thus streamlining extract/strain prioritization and the evaluation of culturing conditions. These capabilities are complemented by an ever-growing collection of public libraries, which includes more than 80,000 MS/MS spectra and allows the fast dereplication of a wide range of NP directly from MS/MS data without the need to perform any fractionation steps. GNPS is continuously growing due to research community data contributions, and it is constantly improving its solutions/informatic tools for data analysis performance, as described below in the reported studies. Having unparalleled capabilities to build MN on MS/MS fragmentation data, together with the possibility to associate metadata such as biological activity and genomics data with the analyses, has revolutionized the NP discovery field. 

GNPS Dashboard enables one to explore the GNPS functionalities; it is compatible with file formats .mzXML, .mzML, .CDF, and raw formats. Analysis and visualization with this tool permitted the creation of URL links and QR codes to promote data sharing [128]. 

MassBank (http://www.massbank.jp (accessed on 23 March 2023) and http://massbank.eu/MassBank/ (accessed on 23 March 2023)) has been a source of data for open libraries, such as GNPS and Human Metabolome Database (HMDB) libraries, MetaboLights, the National Institutes of Standards and Technology (NIST) spectral library, and the MassBank of North America (MoNA; http://mona.fiehnlab.ucdavis.edu/ (accessed on 23 March 2023)). The mzCloud (https://www.mzcloud.org/ (accessed on 23 March 2023)) library contains spectra generated from the same raw data that were used to create MassBank records. The disadvantage is that a spectrum that corresponds to a specific NP across the different databases can have different names and accession numbers due to inter-crossing complexity. Inspired by chemoinformatics InChIKeys, which encode the skeleton, stereochemistry, and charge of the compounds, SPLASH (SPectraL hASH; http://splash.fiehnlab.ucdavis.edu/ (accessed on 23 March 2023)) codes consisting of three alphanumeric blocks were developed to assign unambiguous, database-independent spectrum identifiers that mitigate the previously outlined issue. SPLASH has been implemented in MassBank, MoNA, GNPS, HMDB, MetaboLights, and mzCloud, as well as in the software tools including MZmine, MSDIAL, RMassBank, BinBase, Bioclipse, and the Mass Spectrometry Development Kit (MSDK; https://msdk.github.io/ (accessed on 23 March 2023)) [129]. Open access Monoterpene Indole Alkaloid Database (MIADB), comprising MS/MS data, is available from MetaboLights under the identifier: MTBLS142 (https://www.ebi.ac.uk/metabolights/MTBLS142 (accessed on 23 March 2023)) [130] and was uploaded to the GNPS platform [131]. GNPS analysis combined with the LipidXplorer database has been proposed as an effective approach for assisting structure elucidation and expanding the identification rate of compounds in dereplication studies. By merging the results from both tools and performing a network visualization in Cytoscape, 30 glycoalkaloids were identified in *Solanum pseudoquina* [132]. Moreover, SistematX, available at (http://sistematx.ufpb.br (accessed on 23 March 2023)) is a web-based repository for secondary metabolite data storage and management [133].

Additional assistance in metabolite identification can be provided by MS/MS-Chooser, which automates the creation and uploading of MS/MS reference spectra in GNPS. By enabling rapid data acquisition and analysis (selection of MS/MS spectra), this workflow aids in building public MS/MS spectral libraries, thereby improving and reinforcing annotation tools [134].

The MASST tool (mentioned in bioactivity screening Section 2.2) makes MS/MS searches easier and promotes the reuse of previously reported spectral data, such as public small molecule MS data and environmental and clinical MS datasets. A search engine for public data can be found in MASST (available at https://proteosafe-extensions.ucsd.edu/masst/ (accessed on 23 March 2023)), which offers access to several repositories and libraries and enables users to search a single MS/MS spectrum against public GNPS spectral libraries and all public MS/MS datasets [67].

In GNPS/MassIVE, an online repository accessible at (https://massive.ucsd.edu/ (accessed on 23 March 2023)), all public data are made MASST searchable, including GNPS user-contributed spectra, GNPS libraries, all three MassBanks, ReSpect, MIADB/Beniddir, Sumner/Bruker, CASMI, PNNL lipids, Sirenas/Gates, EMBL, MCF, and numerous other libraries accessible at https://gnps.ucsd.edu/ProteoSAFe/libraries.jsp (accessed on 23 March 2023) [67]. Though molecules with nearly identical fragmentation patterns, such as isomeric metabolites, cannot be distinguished by MASST searches, an original metabolite standard and the use of an orthogonal property (such as retention time) are required. Besides MS/MS spectra search with MASST, the GNPS/MassIVE is a repository for untargeted MS/MS data with sample information (metadata) and annotated MS/MS spectra that can be searched using controlled vocabularies and annotations (ReDU). In 2021, GNPS and the integrated metabolomics data repository MassIVE included 1800 public datasets (>490,000 MS files and >1.2 billion MS/MS spectra), and with over 300,000 visits per month by users from 160 countries, it is one of the most popular MS/MS spectra repositories [135]. Besides MASST, many other analytical tools connected to the GNPS enable direct matching of data to all public MS/MS reference libraries for annotation and MN, thereby facilitating the identification of known metabolites and new derivatives (analogues) of these, as well as fully unknown metabolites and their molecular families. This obviously not only increases the rate of annotation but also helps unearth the real chemical inventory of natural extracts [136]. Moreover, the GNPS infrastructure gives users the power to update annotations in public spectral datasets provided by diverse users while continuously recording all changes [137]. GNPS datasets can also be supplemented with microbiome-related metadata since the software tools used to analyze microbiome data, such as QIIME 2 [138] and Qiita [139], are compatible with the metadata formats used by GNPS/MassIVE. Additionally, by providing a global context to their data and making use of an easier-to-use quick start infrastructure (https://gnps-quickstart.ucsd.edu (accessed on 23 March 2023)), MASST and ReDU enable researchers to control the information in the entire GNPS/MassIVE repository. According to Leão et al., the output from GNPS can also be imported into other analysis programs such as Cytoscape, Metaboanalyst, or QIIME 2, which offer interactive network, statistical, machine learning, or multivariate analysis and visualization capabilities [135]. The above-mentioned software, Qiita, is a web tool that aggregates multi-omics data on microbiome function and composition, enabling meta-analysis and comparison of microbiomes across biospecimens and data layers [139]. 

GNPS users can take advantage of a multitude of additional tools, including: (1) Lickety-split Ligand-Affinity-based Molecular Angling System (LLAMAS), a platform for NP identification and dereplication of DNA-binding molecules from complex mixtures. It uses ultrafiltration-based LC-PDA-MS/MS-guided DNA-binding assays integrated also with Dictionary of Natural Products (DNP), and SciFinder [140]; (2) ConCISE (Consensus Classifications of In Silico Elucidations) establishes accurate putative classifications for entire subnetworks by combining MN, spectral library matching, and in silico class predictions [141]; (3) Spectrum_utils is an open access tool, available at https://github.com/bittremieux/spectrum_utils (accessed on 23 March 2023) for combined and standardized MS data processing and visualization of metabolomics and proteomics data in Python [142]; (4) MetEx, is an open access application, available at https://mo.princeton.edu/MetEx/ (accessed on 23 March 2023), which is suitable for the analysis and visualization of LC-MS metabolomics data of microbial cultures grown under hundreds of elicitors and conditions, facilitating the detection of elicitors/conditions inducing the biosynthesis of several novel and cryptic secondary metabolites [143]; and (5) MetCirc, is a tool for metabolites dereplication that is based on the alignment and comprehensive calculation of pairwise similarities between MS/MS spectra [144]. 

Additional in silico MS/MS approaches, e.g., SIRIUS [145], CSI:FingerID [146], and DEREPLICATOR [147], were also integrated in the GNPS community library. Compatible with GNPS, DEREPLICATOR is an algorithm that allows high-throughput PNP identification. This approach is capable of identifying one order of magnitude more PNPs (and their new variants) than any previous dereplication efforts [147]. DEREPLICATOR+ further improves identification by extending its applicability to polyketides, terpenes, benzenoids, alkaloids, flavonoids, and other classes of NP. Moreover, it also allows cross-validation of genome mining and peptidogenomics/glycogenomics data [148]. NRPro is a MS/MS analysis platform for PNP dereplication and annotation that comprises main functionalities such as automatic peak annotation or statistically validated scoring systems to support the characterization/identification processes [149]. 

In contrast, VarQuest was developed for the identification of PNPs by illuminating the connected components in a MN even if they do not contain known PNPs and only contain their variants. VarQuest discloses an extra order of magnitude of PNP variants when compared to all the previous PNP research efforts. Differing from the ‘comparative metabolomics’ postulation, two related bacteria are unlikely to produce identical PNPs (even though they are likely to produce similar PNPs), which challenges the utility of GNPS for PNP identification [150].

Unlike proteomics, in which optimum acquisition parameters are well described, optimum parameters are not available for generating reliable metabolomic data for MN analysis on the GNPS. Olivion et al., 2017 established an effective system (for Agilent Technologies instruments), simplifying the dereplication process by clearly distinguishing isobaric isomers eluted at different retention times, annotating the MN with chemical formulas, and providing acceptance to semi-quantitative data [151]. 

### 4.3. Dereplication Using MS or MS/MS Advanced Computational Prediction Tools

**Figure 2:**
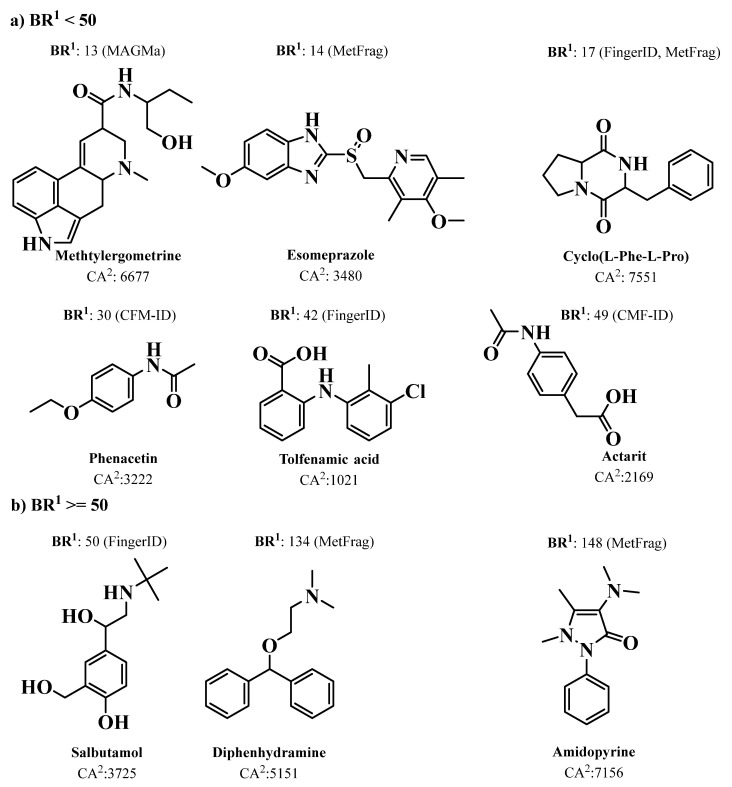
The chemical structures of nine compounds that were correctly identified in the PubChem database by the CSI:FingerID method, but not by any of the other mentioned methods. Where: ^1^ is the best rank achieved by any method but CSI:FingerIDCA; ^2^ is the number of candidate structures in PubChem with the given molecular formula; (**a**) BR < 50 and (**b**) BR ≥ 50.

Over the last ten years, several new approaches have been reported for MS prediction of small molecules that rely on established computational methods such as combinatorial optimization (MetFrag [152], MetFusion [153], MAGMa [154], MIDAS [155], and FT-BLAST [156]) and machine learning (ISIS [157], FingerID [158], CFM-ID [159], and CSI:FingerID [146]) techniques. The emergence of new tools for the prediction of spectral data enabled the development of advanced MS-based dereplication methodologies that clearly translated into a significant improvement in the process of drug discovery from natural sources, including marine biosources. Most of the above MS-based prediction methods, MetFrag, MetFusion, MIDAS, ISIS, FingerID, CFM-ID, and CSI:FingerID, are not supported by spectral library searching; instead, they rely on more comprehensive molecular structure MS/MS database searching. Dührkop et al. developed CSI: FingerID for searching a molecular structure database using MS/MS data. In this workflow, the molecular properties of the unknown molecules are predicted by combining computation and comparison of fragmentation trees with machine learning techniques, linking MS/MS data to open access chemistry databases of molecular structures [146,160]. Significantly increased identification rates were reported for CSI:FingerID when compared with all the existing state-of-the-art metabolite identification tools, such as FingerID, CFM-ID, MAGMa, MIDAS, and MetFrag. In fact, 150% more accurate identifications were achieved by CSI:FingerID than the second-best search method, FingerID. A comparison of prediction performance on a GNPS dataset of 3868 compounds showed that CSI:FingerID reached 5.4-fold more unique identifications compared with the runners-up FingerID and CFM-ID methods, while it correctly detected nine compounds that could not be identified by any other method [160] (Figure 2).

Recently, Dührkop et al. launched SIRIUS 4 (https://bio.informatik.uni-jena.de/sirius/ (accessed on 23 March 2023)), a Java-based software framework for the analysis of LC-MS/MS data of metabolites and other “small molecules of biological interest” [145]. More recently, this platform integrated a collection of computational MS tools that were integrating CSI:FingerID [146] with Confidence Of Small Molecule IdentifiCations (COSMIC) workflow, which performs high-confidence spectral library searching and metabolite annotation of previously unknown structures [161].

**Figure 3:**
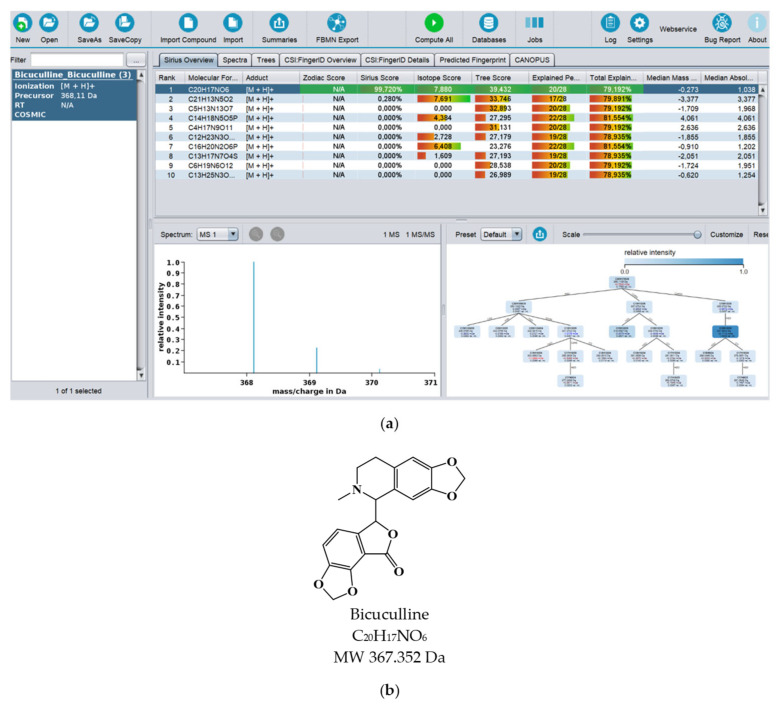
(**a**) Print screen of the SIRIUS 4 software computing the MS spectrum of bicuculline. (**b**) Chemical structure of bicuculline.

In recent years, there has been great development of platforms that integrate various computational MS tools relying on molecular structure database searching, such as ZODIAC (Zero-One Data: Ideal seed Algorithm for Clustering), a network-based algorithm for de novo molecular formula annotation that enables ranking novel molecular formulas that are not present in the most comprehensive public structure databases [162]. CANOPUS (Class Assignment and Ontology Prediction Using Mass Spectrometry), a software for classifying unknown metabolites according to fragmentation spectra using HR-MS/MS data [160], and NPClassifier, a deep-learning neural network-based NP structural classification tool that automatically classifies NP-counted Morgan fingerprints, thus providing the NP structures of their underlying assets [163]. In the same way, SIRIUS 4, mentioned above, combines high-resolution isotope pattern analysis and fragmentation trees with structural elucidation, providing a robust assessment of molecular structures from MS/MS data for big data [145]. The GUI interface of the SIRIUS 4 software is presented in Figure 3. Its users can analyze full LC-MS datasets rather than just one spectrum at a time, and in this way, MS-oriented annotations can be obtained for all the detected resources and not just for those that passed a preliminary statistical test. In fact, SIRIUS 4 achieved reported identification rates of more than 70% on challenging metabolomics datasets [145,146].

Computational MS methods for small molecule annotation have evolved greatly in recent years, as demonstrated by the Critical Assessment of Small Molecule Identification (CASMI) contest (www.casmi-contest.org (accessed on 18 January 2023)) that was held in 2016 [164,165]. One of the challenges of this contest included the determination of molecular formulas using the Seven Golden Rules, Sirius 2, and MS-FINDER software, which were queried in various NP databases, including DNP, UNPD, ChemSpider, and REAXYS, to obtain the molecular structures. To rank these metabolites, a variety of in silico fragmentation tools, such as CFM-ID, CSI: FingerID, and MS-FINDER, were used [164]. Another challenge was the annotation of 19 NP peaks detected across 16 LC-HR-MS/MS profiles. For the purposes of calculating in silico fragmentation and using the molecular formula, XCMS, IPO, RMassBank, CAMERA, and MeHaloCoA tools were used, and two additional external tools, SIRIUS 3 and CFM-ID, were also integrated [166]. The tool MeHaloCoA (Marine Halogenated Compound Analysis) incorporates a mathematical filter based on mass isotopic profiles that allows the selective detection of halogenated (Cl and Br) molecules [167].

Several achievements and pitfalls were revealed from this contest, and valuable conclusions were drawn, such as the anticipation that improvements to machine learning approaches will continue to be introduced as more training data of high quality and annotations become available, whereas chemistry-focused developments such as MS-FINDER will continue to be essential, especially to cover cases where no training data are available [164]. However, several challenges remain. As simple combinatorial optimization approaches such as MetFrag and MAGMa have shown better performance, it is expected that the improved incorporation of experimental data, metadata, will improve the success of annotations, especially in the context of big data [164]. 

### 4.4. Dereplication Using Gas Chromatography-Mass Spectrometry (GC-MS), LC-MS Integrated Ion Mobility Spectrometry (IMS), and LC-Matrix Assisted Laser Desorption/lonization Mass Spectrometry MALDI-MS

A study by Carnevale Neto et al. recently showed that dereplication of NP using GC-MS-based methods can be significantly improved when combining the optimized AMDIS (Automated Mass Spectral Deconvolution and Identification System) software with the RAMSY (Ratio Analysis of Mass Spectrometry) deconvolution tool [168]. Though metabolite identification using GC-MS data will continue to require more caution, many NP are not volatile enough to be analyzed by GC. Furthermore, the high temperatures typically used in the inlet, column, and ion source of a GC (>300 °C) often decomposes NP or cause their structural rearrangement.

The GNPS dashboard also enables one to explore the functionalities related to the dereplication methods described below. The MS data repositories include GNPS/MassIVE, MetaboLights, ProteomeXchange, and Metabolomics Workbench, as well as data from proteomics resources: PRIDE and MassIVE [128,169,170].

To facilitate the analysis of GC-MS data and metabolite annotation, the MSHub machine-learning deconvolution tool was deployed within GNPS. With this approach, the compound fragmentation patterns are auto-deconvoluted via unsupervised non-negative matrix factorization, and the reproducibility of deconvoluted fragmentation patterns across samples is quantified, providing a measure of de-convolution performance [171]. 

Marshall et al. suggested Integrating Ion Mobility Spectrometry (IMS) as a valuable NP dereplication tool [172]. As extract complexity defies the resolving power of modern LC-MS/MS pipelines, by using IMS with LC-MS/MS, both metabolite detection and the quality of MS/MS spectra are improved. This is because IMS provides an additional separation that is orthogonal to chromatographic and mass spectral separations. The IMS technique separates the ions in the gas phase and enables the measurement of their rotationally averaged Collision Cross-Section (CCS), which is an important distinguishing characteristic for identification purposes. The effect of integrating IMS in LC-MS/MS for the characterization of NP was recently evaluated on MS/MS fragmentation data of an actinobacterial extract spiked with 20 commercial standards using both Data-Dependent Acquisition (DDA) and Data-Independent Acquisition (DIA). Examining those datasets in the GNPS platform revealed that the inclusion of IMS increased both spectra quality and metabolite detection, particularly for samples analyzed in DIA mode [173]. 

Matrix Assisted Laser Desorption/lonization Mass Spectrometry with Time-of-Flight detector (MALDI-TOF-MS) can be used for efficient dereplication of microbial isolates, including their taxonomic identification and characterization, for downstream studies with negligible loss of unique organisms. The dereplication performance of whole-cell MALDI-TOF MS-based analyses and 16S rRNA gene sequencing was compared using 49 bacterial cultures, and both methods were found to yield comparable taxonomic assignments up to the genus level [174]. The agreement of the methods at the species level was limited, which was attributed to the small mass spectral reference databases, though the latter can be significantly improved in the future, unlike 16S rRNA gene analysis, whose methodological limits have reached a plateau. Moreover, the MALDI-TOF MS technique was deemed to provide superior resolution than 16S rRNA gene analysis, as it can better distinguish bacteria with very high 16S rRNA similarity (i.e., > 99.2%). Besides the dereplication of bacterial isolates, MALDI-MS can also enable the rapid and comprehensive profiling of NP mixtures. In particular, with the provision of biosynthetic heavy-isotope-labeled precursors, MALDI-MS can be a powerful method for dereplication and identification of unique metabolites. The power of this approach was exemplified with the detection/characterization of cryptomaldamide and several new peptides of the viequeamide class in a marine cyanobacterium [175].

SpeDE is an algorithm available at https://github.com/LM-UGent/SPeDE (accessed on 23 March 2023), which enables the rapid dereplication of microbial isolates resulting from clinical or environmental studies through the dereplication of their MALDI-TOF mass spectra. Βeing capable of identifying sets of similar spectra at the species level, this tool exceeds the taxonomic resolution of other methods and effectively helps minimize the number of redundant isolates. Given its high speed and accuracy, the SpeDE algorithm streamlines the culturomics approach to bacterial isolation campaigns [176].

Mass spectrometry imaging for two- (2D) or three-dimensional (3D) molecular visualization of biological structures is becoming increasingly popular by leveraging the unique analytical advantages offered by MALDI-MS and DESI-MS (Desorption Electrospray Ionization) systems [177,178]. Owing to the sheer quantity of data generated, the visualization, analysis, interpretation, storage, and sharing of 3D imaging MS data remains a significant challenge. MetaboLights can handle the large mass spectrometric datasets produced from the 3D imaging of biospecimens, such as tissue sections, entire organs, or microbial colonies [179,180]. 

A schematic representation of the existing methodologies for MS/MS, GC-MS, IMS, and MALDI dereplication is presented in Table 1. 

### 4.5. Dereplication Using NMR Spectroscopy

Although MS/MS is much more sensitive, NMR is more robust and accurate. NMR-based dereplication databases have also evolved over the last few years, from strategies that employed calculated/real NMR data or structural features easily recognizable in 1D NMR spectra to those that employed 2D NMR data. Advances in NMR include pulse sequences for molecular structural characterization of isolated compounds, ^13^C NMR metabolomics platforms for screening NP libraries, and miniaturization via microNMR spectroscopy [181]. The structural properties that define ^13^C NMR signals as characteristic representations of a given molecule are the chemical shifts (δ in ppm) and coupling constants (*J* in Hz), along with the line widths (Δν in Hz). These parameters are bound both to the molecule and the NMR experimental conditions by quantum mechanical (QM) principles. During the development of the HiFSA (^1^H Iterative Full Spin Analysis) method for preventing structural misassignments of NP, Pauli et al. highlighted the importance of submitting FID (Free Induction Decay) files with publications and in databases to support advances in NMR dereplication and structure elucidation [182]. The power of NMR for structural elucidation of NP has been illustrated in numerous studies, including the rigorous characterization of several novel peptides from the viqueamide class that were isolated from a marine cyanobacterium [175]. A fragment-based strategy relying on digital ^1^H NMR profiles generated by HiFSA has been developed for dereplicating structurally related molecules that have the same carbon skeleton but different numbers of substituents and/or substitution patterns [183]. In this approach, digital representations of known structural motifs are generated and subsequently combined as building blocks to facilitate the interpretation of ^1^H NMR spectra of increasingly complex molecules [183]. 

NMR analysis is a powerful complement to MS approaches, providing useful data sets in a reasonable time frame. However, the high degree of signal overlap, particularly in 1D NMR spectra, combined with the insufficient precision in NMR spectroscopic analysis and the rationality in reporting Δδ and ΔJ values limit the applications of this approach in high-throughput dereplication [184]. The low sensitivity of NMR is another limitation, but ^13^C NMR has many advantages for dereplication, such as its universal detection capacity, which enables simultaneous high-resolution analysis of any organic compounds, and its ability to distinguish structurally close NP, including stereoisomers [185,186]. In this context, the MixONat algorithm was developed in Python for ^13^C NMR-based dereplication. It analyzes ^1^H-^13^C NMR spectra with the options to apply molecular weight (MW) filtering and to take into account DEPT-135 and DEPT-90 data for distinguishing different carbon types (i.e., CH_3_, CH_2_, CH, and C), which can help improve dereplication performance [185,186]. 

A computer-aided ^13^C NMR-based dereplication method was reported by Bakiri et al. for the metabolite profiling of NP extracts without any fractionation [187]. By comparing the ^13^C NMR chemical shifts of the crude extract with those predicted from database records, the algorithm calculates matching scores and creates a list of metabolites that are most likely to be present. In another study, one-dimensional ^13^C NMR data and machine learning methods were employed to develop the XGBoost classifier, which predicts the chemical class of NP with higher accuracy, outperforming other algorithms of the same type [188].

Several informatics tools have been developed for comparing 2D NMR spectra with libraries of reference spectra to dereplicate NP and determine molecular structures. However, spectroscopic artifacts, solvent effects, and the interactive effect of functional group(s) on chemical shifts hamper the efficiency of this approach [184]. To simplify spectral analysis and accelerate chemical identification of components in complex mixtures, the 2D NMR barcoding methodology was developed. It uses the molecular information from NMR spectra (i.e., ^1^H-^13^C correlation signals and their spatial locations in the δ_H_−δ_C_ coordinate space) to generate 2D barcodes that facilitate dereplication by in silico matching of experimental and reference barcodes to facilitate the chemical identification of complex mixtures [189].

In 2018, Bakiri et al. developed a 2D NMR-based method for the dereplication of metabolite mixtures that relied on the combination of Heteronuclear Multiple Bond Correlation (HMBC) and Heteronuclear Single Quantum Correlation (HSQC) spectra. The latter provides very rich information about short-range and long-range H-C correlations that occur in the carbon skeleton of individual chemical entities. In analogy to molecular networking from MS/MS spectra, this method uses the HMBC spectrum of a metabolite mixture to create the network of ^1^H-^13^C correlations, which is then divided into clusters of correlations using a community detection algorithm, and the clusters are subsequently assigned to specific molecular structures by searching a database containing theoretical HMBC and HSQC correlation data of natural metabolites [190]. A pipeline that integrates GNPS-curated MS/MS data with HSQC and HMBC 2D NMR data using a robust *^n^J_C,H_* network analysis has been developed by Kuhn et al. for enhancing NP identification in complex mixtures. This aimed to exploit the complementary advantages offered by NMR (high reproducibility and efficiency in structure elucidation) and LC-MS/MS (high sensitivity and accuracy) techniques. In this approach, MS/MS-based molecular network dereplication is performed, and the identified candidate structures are ranked according to the probability of being present in the sample by predicting their HMBC-HSQC NMR spectra and comparing them to the measured spectrum of the mixture [191]. Both the prediction of NMR spectra and the matching with the experimental data are performed by the embedded NMR filter algorithm. The specific tool has the capability to identify uncatalogued compounds, and it has been shown to provide comparable results with COLMAR (Complex Mixture Analysis by NMR), which is the leading system for elucidating the components of metabolite mixtures [185]. 

Small Molecule Accurate Recognition Technology (SMART) is another tool that was recently developed to accelerate the discovery and characterization of new NP. This machine learning tool uses an artificial intelligence (AI) algorithm based on convolutional neural networks (CNN) to map the HSQC NMR data of the analyzed mixture or compound into a multidimensional space, which has been formed by a library of 100,000 known molecules with both experimental and simulated HSQC data. In these SMART maps, similar compounds are placed near one another and dissimilar compounds are placed far apart, thus allowing for the revelation of candidate structures for a mixture/secondary metabolite by assessing the spatial position of their queried data in HSQC space [192]. Queries can be performed using .csv, .tsv, TopSpin peak data, or manually entered data, whereas the biological context of the results is aided by the provision of external links to Natural Products Atlas [92], MIBiG [193], and GNPS [98] in the case of known NP.

SMART-Miner is also a convolutional neural network-based tool that uses ^1^H-^13^C HSQC spectral data for NP identification. This method performed accurate identification of individual metabolites with higher peak intensity or similar chemical shifts from different metabolites, which is a drawback, but it presented higher performance when compared with other NMR-based metabolomic methods [194].

SMART 2.0 was launched for the analysis of extracts using marine cyanobacterium *Symploca* with the aid of MS/MS-based MN, leading to the fast identification of a new chimeric swinholide-like macrolide, symplocolide A, as well as the annotation of swinholide A, samholides A-I, and several other novel derivatives. Another example was the use of SMART 2.0 for the characterization of novel cyclic peptides, demonstrating the groundbreaking potential of combined traditional and deep learning-assisted analytical approaches to overcome old challenges in NP lead discovery [192,195].

MatchNat is another in silico tool that was specifically developed for the 2D NMR-based dereplication of diterpene alkaloids (DAs) in complex mixtures. This dereplication strategy is based on heteronuclear multiple bond correlation (HMBC), and it utilizes the characteristic HMBC patterns provided by the majority of C_19_-DAs as diagnostic signals for recognizing already known compounds and identifying novel DAs [196]. In this context, MatchNat performs an automatic comparison of experimental NMR data from complex mixtures with those of a reference database consisting of approximately 350 natural C_19_-DAs [196]. 

Another example of new developments in NMR-based dereplication methodologies is DEREP-NP (freely available at https://github.com/clzani/DEREP-NP (accessed on 23 March 2023)), which is applicable to purified natural products or fractions containing a small number of compounds. This platform generates a database containing counts for 65 structural fragments present in the >220,000 NP of the Universal Natural Products Database (UNPD), while inferring the counts of the same fragments in an unknown compound from its NMR spectrum (^1^H, HSQC, and/or HMBC). The latter data are used to create a numeric combination, which is searched against the database in order to retrieve candidate structures [197].

A recently released approach uses Diffusion-ordered NMR spectroscopy (DOSY) to dereplicate NP in mixtures of compounds [198]. This technique enables accurate measurement of the diffusion coefficient (*D*) for the different mixture components, which is mainly related to MW. The same parameter can be accurately predicted for any compound present in the DEREP-NP database using a multiple linear regression model that involves eight structural and chemical properties, including molecular weight. By matching experimental *D* and structural features derived from NMR analysis with predicted *D* and calculated structural features in the database, the dereplication of known NP in a mixture can be achieved. On the other hand, the absence of hits from database searches can be used to track down new compounds [198].

Diaz-Allen et al. suggested that 1D-Total Correlation Spectroscopy (1D-TOCSY) offers unique capabilities for NP dereplication, as it allows not only to detect known compounds but also to identify possible new structures in a mixture that are structurally related to known compounds in a TOCSY library [199]. In another study, a pipeline combining data from GC-MS and NMR analysis with the use of Statistical Total Correlation (STOCSY) spectroscopy was developed to achieve higher confidence in compound identification [200]. Moreover, MADByTE (Metabolomics and Dereplication by Two-Dimensional Experiments) is another platform that was developed for the dereplication of known compound scaffolds and the prioritization of bioactive metabolites from prefractionated extracts [201]. By combining TOCSY and HSQC spectra, it identifies spin system features within complex mixtures and then matches spin system features between samples to create a chemical similarity network for a given set of samples. Unlike many of the existing NMR-based profiling tools, it does not require a bespoke spectral reference library against which to compare NMR data. However, the use of a database of pure compounds with MADByTE is also possible, and it is particularly helpful when the dereplication of specific compound classes (e.g., resorcylic acid lactones, spirobisnaphthalenes) is of interest [201,202].

#### 4.5.1. NMR and NP Databases for Dereplication

Regarding databases containing structural information on MNP, since its initial development as an in-house developed system in the 1970s by Profs. Munro and Blunt from the University of Canterbury, MarinLit (https://pubs.rsc.org/marinlit/ accessed on 23 March 2023) stands as one of the most useful tools in marine NP dereplication. The database is currently maintained by the Royal Society of Chemistry and, through a recently launched web interface, offers comprehensive coverage of more than 37,000 articles on MNP. Searching the database for dereplication purposes offers multiple possibilities using any combination of substructure, NMR structural features obtained from direct interpretation of spectra, calculated ^13^C and ^1^H NMR shift data, exact mass, chemical formula, UV λ_max_, and log Ɛ. It is linked to taxonomy, and full references to publications describing the molecules are also provided.

The Natural Products Atlas, available at (www.npatlas.org accessed on 23 March 2023), was created in 2019 and emerged as a comprehensive database covering all microbially-derived NP published in the peer-reviewed primary scientific literature [196]. Its initial version covered more than 25,000 microbial compounds and contained referenced data for structure, substructure, compound names, source organisms, isolation references, total syntheses, physical properties, author, discovery timeline data, and instances of structural reassignment. This open access community-supported repository was established under FAIR principles (Findable, Accessible, Interoperable, and Reusable), and it is combined with other NP databases, including the Minimum Information about a Biosynthetic Gene Cluster (MIBiG) repository and the GNPS platform [92]. This database has been updated in 2022 to The Natural Products Atlas 2.0, including a full RESTful application programming interface (API), a new website framework, and was expanded in terms of metabolites, including 8128 new compounds, bringing the total to more than 32,000 [203]. Full taxonomic descriptions for all microbial taxa and chemical ontology terms from both NP Classifier and ClassyFire were added; configurational assignments were revised; and data from external resources was also added, including the integration of CyanoMetDB [203,204].

NP-MRD (The Natural Products Magnetic Resonance Database) is an open access NMR repository, available at https://np-mrd.org accessed on 23 March 2023, supporting community deposition of NMR meta-data assignments and NP NMR spectra (1D and 2D) [205].

The StreptomeDB 3.0 includes a compendium of more than 6000 NP produced by actinomycetes [206]. Apart from structures or substructures, NMR and/or MS/MS data can be used as input in searches of the database for dereplication purposes. It also enables the interactive phylogenetic exploration of *Streptomyces* and their isolated or mutasynthesized NP, being the only public online database offering this functionality. The entries in this database are hyperlinked to several spectral, (bio)chemical, and chemical vendor databases and to MIBiG. Moreover, prediction methods for ADMET profiling are available. Finally, structures combined with metadata can be downloaded in SD-Format, allowing their incorporation into other structural features of NP dereplication tools such as DEREP-NP. 

The COlleCtion of Open NatUral producTs (COCONUT), an open access collection of NP launched in 2020, is one of the biggest resources for NP annotation. The database includes structures of more than 400,000 unique NP (without stereochemistry) that can be extended to more than 730,000 when stereochemical variants are taken into consideration [13]. It offers interesting functionalities such as predicted bioactivities, molecular descriptors, known stereochemical variants of each entry, and bibliographic references. As in the case of StreptomeDB 3.0, the full set of structures can be downloaded in SDF or SMILES format, allowing their use in combination with other structural feature-based databases for dereplication purposes [13].

More recently, in 2021, Lianza et al. proposed two NMR-based tools: the Predicted ^13^C NMR data of Natural Products (PNMRNP) database, which originates from UNPD, and KnapsackSearch, a database generator that provides taxonomically focused libraries of NP [91].

The Comprehensive Marine Natural Products Database (CMNPD) is an open access database (available https://www.cmnpd.org accessed on 23 March 2023) that includes information on 31,000 marine-derived chemical entities. By providing a plethora of data, such as physicochemical and pharmacokinetic properties, standardized biological activity data, systematic taxonomy, geographic distribution of source organisms, and detailed literature citations, it aims to facilitate structure dereplication, the discovery of lead compounds, data mining of structure-activity relationships, and the study of chemical ecology [207]. 

Natural Products and Biological Sources (NPBS) is a repository of NP chemical data that correlates NP with their biological sources, a feature that is not available in all the databases [152].

A schematic representation of the existing methodologies for NMR dereplication is presented in Table 2.

When comparing the existing LC-MS/MS, GC-MS, LC-IMS, and LC-MALDI-MS dereplication tools (Table 1; Section 4.4) with the LC-NMR dereplication tools (Table 2), it is emphasized that the methods for dereplication using MS are far more developed than for NMR. Further research on NMR methods, especially when integrated with MS/MS methods, would increase dereplication efficiency and accuracy.

## 5. Genome Sequencing Methods for Dereplication and Structure Elucidation

NP are produced by biosynthetic enzymes that build core scaffolds or carry out peripheral changes and can be defined as NP families, introducing pharmacophores and allowing metabolic diversity. Our capacity to access and characterize NP pathways using sequence-similarity-based bioinformatic tools has been substantially improved by contemporary genomics approaches [208]. Rapid and low-cost genome sequencing, as well as the development of bioinformatical analysis tools for biosynthetic gene cluster identification in conjunction with MS-based molecular networking, aided in the process of dereplication [22]. Fascinating cases of unique enzymology have been recently discovered, supporting NP structure elucidation through the annotation of NP biosynthetic pathways. Nevertheless, several biosynthetic enzymes that catalyze amazing and unique reactions continue to challenge functional prediction and remain hidden from (meta)genomic sequence data [208,209]. The development of next-generation sequencing (NGS) and the emergence of potent computational tools are starting to expose previously unrecognized taxa, ecological niches, and “biosynthetic dark matter”, connecting phenotype and chemotype and revealing a variety of NP that are diverse and chemically distinct in previously unstudied microorganisms [50,84,210,211].

### 5.1. Genome Sequencing Techniques

From 1977 to 2022, four generations of sequencing technologies have been developed, offering many advantages over classical Sanger sequencing, referred to as the first generation sequencing (FGS), where the terminator ddNTP is tagged with specific fluorescent dyes [212]. 

Since their inception in 2004, the second (SGS) and third generation sequencing (TGS) technologies, commonly referred to as next-generation sequencing (NGS) technologies, have undergone tremendous development with a rise in sequencing speed and a decrease in sequencing cost. There are several different types of sequencing platforms for SGS, starting with GS FLX by 454 Life Sciences/Roche Diagnostics (2004), Genome Analyzer (2006), HiSeq, MiSeq, and NextSeq (2015) by Illumina, Inc., SOLiD by ABI (2007), and Ion Torrent by Life Technologies (2010), which differ in sequencing chemistry that leads to differences in throughput, read length, genome coverage, error rate, cost, and run time [213]. Two main steps common to all SGS involve template preparation (nucleic acid extraction, library preparation, and amplification), followed by sequencing, which comprises two main approaches: (1) sequencing by synthesis (SBS) and (2) sequencing by hybridization and ligation (SHL). Three main classes of sequencing chemistry in SBS include pyrosequencing, sequencing by reversible termination (Illumina), and sequencing by detection of hydrogen ions (Ion Torrent) [214]. The main limitation of pyrosequencing, based on the detection of pyrophosphate (PPi) during DNA synthesis, was inaccurate sequencing of homopolymers since the addition of more than five identical nucleotides could not be accurately detected [215]. Illumina sequencing platforms allow paired-end sequencing as DNA fragments of the libraries are subjected to clonal amplification by bridge PCR [216], followed by sequencing using reversible terminator (RT) nucleotides. Here, the homopolymer sequencing error is overcome by adding a single base at a time with the terminator removed from the previous base. In addition to resulting in high readings and coverage compared to the sequencing system at one end, sequencing a DNA fragment at both ends also greatly facilitates the detection of genomic rearrangements, repetitive sequences, gene fusions, and novel transcripts. In addition, Illumina platforms provide superior alignment across DNA regions containing repetitive sequences and generate longer contigs for de novo sequencing by filling gaps in the consensus sequence [214]. Sequencing by detection of hydrogen ions (Ion Torrent sequencing, pH-mediated sequencing, silicon sequencing, or semiconductor sequencing) is another SBS method based on the detection of hydrogen ions that are released during DNA polymerization and applicable for whole-genome sequencing and RNA-Seq. Both Illumina and Ion Torrent platforms provide alternative approaches for studying RNA at the sequence level, have similar capacities, and may be used to examine different transcriptional phenomena through careful selection of the software alignment [217]. The SOLiD (Support Oligonucleotide Ligation Detection) sequencing platform, which is based on ligation (using DNA ligase) rather than synthesis, although it does not produce long-reading sequences that make assembly more challenging, has remained competitive based on cost per base.

Third generation sequencing aimed to overcome two main SGS limitations: short read length and consequently the need for bioinfomatic pipelines for the sequence assembly, as well as PCR bias as a result of clonal amplification (bridge PCR amplicifation) for the development of a detectable base incorporation signal [214]. The platforms available for the TGS are HelicosTM Genetic Analysis System by SeqLL, LLC; SMRT Sequencing by Pacific Biosciences; and Nanopore sequencing by Oxford Nanopore, as single-molecule real-time technology platforms; as well as Complete Genomics by Beijing Genomics Institute (based on SHL); and GnuBIO by BioRad, a droplet-based DNA sequencing platform that utilizes microfluidic and emulsion technology to perform complex, multiplexed reactions in droplets (2014; Bio-Rad Laboratories, Inc.).

### 5.2. High Throughput Next-Generation Sequencing (HT/NGS) 

HT/NGS technologies can generate massive amounts of data, given their higher sequencing efficiency and lower cost per base. The rough division of HT includes genome sequencing and transcriptome sequencing (RNA-seq). In both cases, the reading may be single or paired ends, while reads that are generated from both ends of longer fragmented DNA or RNA significantly increase the sequence accuracy. Genome sequencing involves the sequencing of fragmented genomic DNA and the assembly of the entire genome from the read sequence. Transcriptome sequencing (RNA-Seq) provides insight into the presence and quantity of RNA sequences in a biological sample in real time by continuously analyzing changes in the cell transcriptome. In addition to information on gene expression at the genome-scale level, it is also possible to measure the expression levels of a smaller subset of genes using this technique [214]. RNA-seq has made an outstanding contribution to elucidating various aspects of RNA biology, including single cell gene expression, translation (the translatome), RNA structure (the structurome), as well as spatial transcriptomics (spatialomics) [215], by becoming the method of choice for transcriptome analysis.

Nanopore-based technologies as fourth-generation sequencing drivers enable HT and provide the longest read lengths, from 500 bp to the current record of 2.3 Mb, with common genomic libraries ranging from 10 to 30 kb [218]. Silicon nanotechnology has really pushed genomics forward, facilitating complex workflows. Thus, nanopores can be integrated into a chip, paving the way for mini-portable DNA sequencing devices. The long-reading sequence, unlike conventional HTS, where the length of the reading sequence is limited to a few hundred nucleotides or less, is rapidly gaining popularity and is likely to completely prevail over other sequencing technologies [219]. Here, each reading can be several thousand nucleotides long, which has several advantages over short-reading technologies. Long-reading technology allows the omission of assembly to obtain whole genome sequences for prokaryotes, while complex splice junction detection procedures can be skipped for eukaryotic transcripts. Pacific Biosciences (PacBio) and Oxford Nanopore Technologies (ONT) are the two key competitors driving innovation in this technology.

The quality of the genome sequence is crucial for secondary metabolite biosynthetic gene cluster (smBGC) identification, significantly facilitating functional gene annotation. Due to the fact that the majority of BGCs consist of core biosynthetic genes, mostly larger than 5 kb and usually containing repetitive sequences, it is obvious that an inaccurate genome sequence often results in frameshift errors during the prediction of coding regions within the BGCs [220]. On the example of the genome sequences of *Streptomyces clavuligerus* ATCC 27064, a Gram-positive bacterium with industrial and clinical significance that produces β-lactam class antibiotics (the β-lactamase inhibitor clavulanic acid), it is notable that the sequence qualities significantly affect BGC identification. Thus, for the first time, *Streptomyces clavuligerus* ATCC 27064 genome sequence was obtained by random shotgun Sanger sequencing using ABI 3700 [221], followed by a draft genome sequence of *S. clavuligerus* NRRL 3585 (ATCC 27064) obtained by a hybrid approach that involved Sanger sequencing and Roche/454 pyrosequencing [222]; and then a high-quality *S. clavuligerus* genome sequence was obtained using PacBio long-reading sequencing and Illumina short-read sequencing methods [223]. The latter genome sequencing of *S. clavuligerus*, which filled all sequence gaps and corrected errors from the previous contig sequences, resulted in a 6.75 Mbp linear chromosome and a 1.8 Mbp mega-plasmid, 7163 newly annotated genes, and 58 smBGCs. Among these, 30 and 28 BGCs were found from the chromosome and the plasmid, respectively, in comparison with 23 and 25 BGCs previously identified by Song et al. [222]. Recently published high-quality genome sequences of 22 *Streptomyces* species and eight strains of *Streptomyces venezuelae* confirmed that assembling by a hybrid strategy, using genome sequencing methods for long reading and short reading, facilitated the detection of new secondary metabolites and the identification of smBGCs [224].

### 5.3. Dereplication Using Genomics Methods 

In the past decade, plenty of new platforms and databases have been developed to computationally mine genetic data and its links to known NP. The use of this approach is exponentially increasing for the discovery of new natural entities. The dereplication strategy using genomic methods derives from the fact that the structures of the enzymes that are involved in the production of NP are amazingly conserved, and so their encoding genes are organized in clusters, known as biosynthetic gene clusters (BGC). These BGCs can be defined as a group of genes in close genomic proximity that together promote the synthesis of NP through a complex route of enzymatic reactions and regulatory switches [225]. Such clusters encode not only proteins that synthesize the final products (backbone enzymes), but also genes encoding potentially regulatory elements such as transcription factors (TFs), transport proteins, resistance factors, or those involved in precursor production [226]. 

The most studied compound classes are polyketides (PK), biosynthesized by polyketide synthases (PKS), and non-ribosomally synthesized peptides (NRP), produced by non-ribosomal peptide synthetases (NRPS), along with ribosomally and post-translationally modified peptides (RiPPs). In particular, NRPS enzymes are very good candidates for genome mining approaches because of their good co-linearity of the modular domain organization with their corresponding biosynthetic products and their high degree of conservation, although there are exceptions to that co-linearity rule [52]. SANDPUMA, an improved tool when compared with prediCAT for the dereplication of NRP chemical space, is available as an open source, and it has been integrated into antiSMASH [227]. NRPminer is a modification-tolerant instrument for NRP discovery integrating large (meta)genomic and MS datasets [228]. Nerpa is a software tool for the high-throughput discovery of novel BGCs that produce NRPS [229].

CycloBranch is an open access cross-platform, available at http://ms.biomed.cas.cz/cyclobranch/ (accessed on 22 March 2023) [230], for annotating spectra of linear, cyclic, branched, and branch-cyclic nonribosomal peptides and polyketide siderophores. MassSpecBlocks converts chemical structures, searchable in public databases such as PubChem, ChemSpider, ChEBI, NP Atlas, COCONUT, and Norine, available in SMILES format, into sequences of building blocks and proteinogenic amino acids. Moreover, it allows the construction of custom sequence and building block databases to annotate mass spectra in CycloBranch software [231].

The iSNAP platform uses an in silico algorithm for screening tandem MS data as an accurate tool for fast dereplication and profiling of large NRPS families [232]. 

These biosynthetic pathways can be computationally predicted and prioritized by genome mining, which allows not only the prediction of the structure of the NP based on genetic information prior to its isolation and structural elucidation by spectral data but also their possible functional and chemical interactions. The main premise of the in silico mining method is the use of multiple sequences that encode the reference enzymes (“core biosynthetic genes”) for the identification of homologues in the genome sequences, allowing the selection of the most interesting biotechnology-based microorganisms. In overcoming the limitations of culturing microbial isolates, improved sequencing and analysis methods have broadened our understanding of the microbial world. 

The availability of published genome sequences of a huge number of microorganisms, along with the development of a plethora of computational tools, has revolutionized strategies to detect and prioritize the search for new NP using gene clusters. Thus, the evolution of sequencing technologies from the classic chain termination method to fourth-generation sequencing resulted in 19,865 complete (7425) and permanent draft (12,440) genomes, as well as 40,583 complete and 23,313 incomplete genome sequencing projects in 2020. Although these numbers were significantly lower in 2021 due to the COVID-19 pandemic, they saw a renewed increase in 2023 (https://gold.jgi.doe.gov/statistics (accessed on 22 March 2023)) [233]. 

Genome mining (GM) comprises computational methods for the automatic detection and annotation of BGCs from genomic data. Moreover, as identification of biosynthetic pathways of NP leads to elucidation of their possible functional and chemical interactions [52], machine learning (ML) genome mining approaches deeply contribute to understanding NP chemical diversity through analysis of microbial and plant genome architecture and structure, or their “BGC genomic language” [29]. Thus, through identification and BGC analysis, GM has become a key technology to exploit and explore NP diversity [234].

GNPS can be linked to genomic information to aid genome-driven NP discovery, with the discovery of columbamides demonstrating this approach [175,235,236]. *Streptomyces tendae* VITAKN isolated from the southern coast of India was dereplicated using integrated genome mining coupled with MS/MS analysis and in silico GNPS tools. The sequence similarity networks of the detected BGCs from this strain against the MIBiG database and 3365 BGCs predicted by antiSMASH analysis of publicly available complete *Streptomyces* genomes were generated through the BiG-SCAPE-CORASON platform to evaluate its biosynthetic novelty. The identification of cyclic dipeptides (2,5-diketopiperazines, DKPs), which are known to possess quorum sensing inhibitory (QSI) activity, was also achieved [237].

Natrix is an open-source bioinformatics workflow available on GitHub (https://github.com/MW55/Natrix (accessed on 22 March 2023)) or as a Docker container on DockerHub (https://hub.docker.com/r/mw55/natrix (accessed on 22 March 2023)) and written using Snakemake for preprocessing raw amplicon sequencing data. This comprises a comprehensive method, from quality assessment, read assembly, dereplication, chimera detection, split-sample merging, sequence representative assignment (OTUs or ASVs), to the taxonomic assignment of sequence representatives. Snakemake guarantees reproducibility, and Conda (https://docs.conda.io/en/latest/ (accessed on 22 March 2023)) controls the applied programs [238].

The Paired Omics Data Platform is a community-led effort to systematically document links between metabolome and (meta)genome data, thereby assisting in the identification of NP biosynthetic origins and metabolite structures [239].

NP are synthesized by biosynthetic gene clusters (BGCs), whose genes are involved in the production of one or a family of chemically related metabolites. Walker and Clardy in 2021 developed a machine learning bioinformatics method for predicting biological activity for genes [240]. Bioactivity prediction can also be achieved through a multiplex genome editing system using a cytosine base editor (CBE) [241].

#### 5.3.1. Retrieving the Microbial/Environmental DNA 

Metagenomics is the process of extracting microbial genomes directly from environmental samples, regardless of sample type or microbial abundance [242]. Metagenomics, as a culture-independent method, utilizes the sequencing revolution to overcome many of the conventional barriers to NP discovery by profiling microbial communities and accessing the biosynthetic capacity of the environmental metabiome. The progression of readily available bioinformatic pipelines has enabled large quantities of BGCs to be mined from environmental microorganisms without having to culture them and test their bioactivity. In addition to identifying new metabolites, metagenomic sequence data assembly led to the identification of the “metabolically talented” endosymbiontic genus *Entotheonella*, which is expressed in almost all bioactive molecules that have been isolated from its host, the marine sponge *Theonella swinhoei* [243]. Culture-independent methods have substantially contributed to our understanding of global microbial diversity. The first large-scale initiative to recover nearly 8000 bacterial and archaeal metagenome-assembled genomes (MAGs) from over 1500 publicly available metagenomes, named the Uncultivated Bacteria and Archaea (UBA) data set, showed the tremendous importance of developing algorithms for the construction of entire genomes from environmental samples and substantially expanded the tree of life [244]. Single-amplified genomes (SAGs) and MAGs are two examples of genome analysis from uncultivated species that have recently contributed to our understanding of microorganisms and additionally contribute to the elucidation of the tree of life.

Furthermore, advances in sequencing technologies have expanded the availability of genomes and metagenomes, significantly facilitating community-wide microbial pan-genome research [245]. In keeping with the current trend, studies on individual microbial genomes and their genotype/chemotype/phenotype relations have increasingly moved from individuals to environmental microbial communities directed towards predicting multiple entities simultaneously. The pangenome concept is based on the fact that “the sequence of a single genome does not reflect the entire genetic variability of a bacterial species” [246]. It can be applied in either a reverse approach with the aim of capturing the genomic diversity of the group of interest or a forward-thinking approach with the aim of estimating the minimum number of genome sequences required to capture the entire genomic repertoire of the group, which should not be less than five [247]. Pangenome analysis may be useful for redefining the taxonomic and pathogenic positions, as already demonstrated on species of the genus *Shigella* and *Escherichia coli* strains [248], but also as a promising tool for identifying novel secondary metabolites through microbial communality profiling. Mohite et al. reported in 2019 the (pan)genome mining of 2627 enterobacterial genomes, which resulted in the detection of 8604 BCGs, corresponding to 212 BGC families, of which only 20 were associated with previously characterized BGCs from the MIBIG database as siderophores, antibiotics, and genotoxins [249]. 

#### 5.3.2. Steps and Tools in Genome Mining

**Figure 4:**
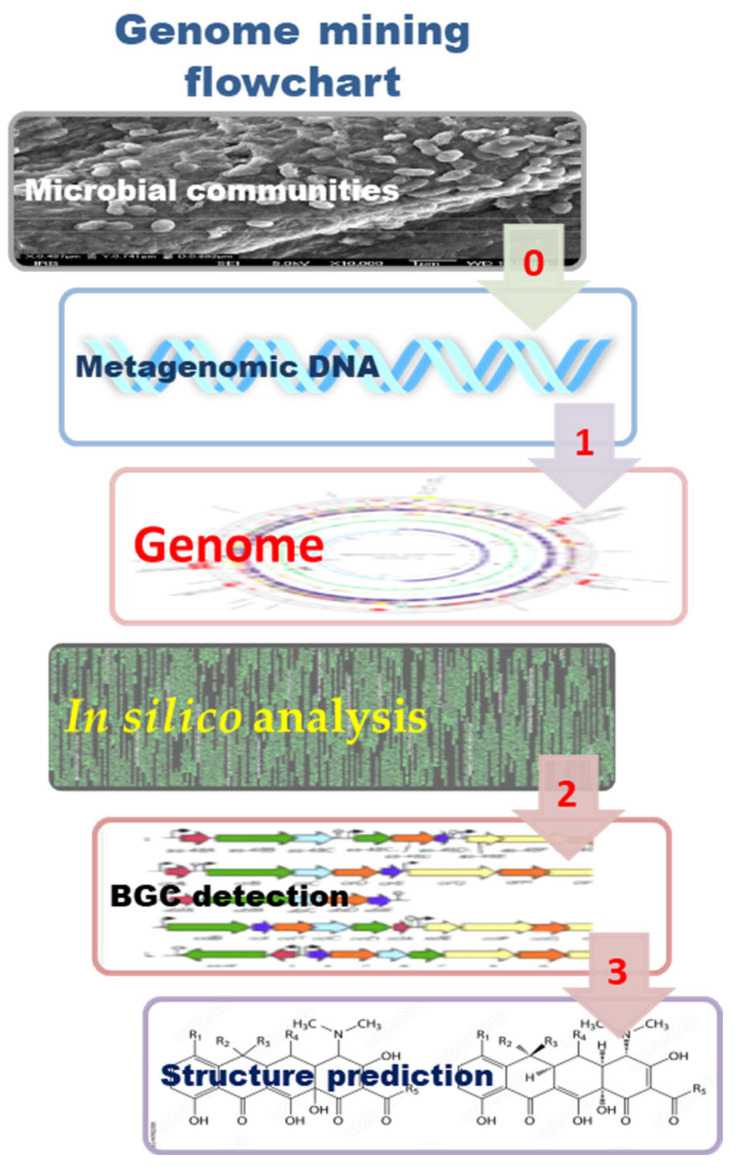
Retrieving microbial/environmental DNA and main steps of genome mining: (1) genome annotation; (2) detection and identification of biosynthetic gene clusters (BGCs); and (3) the prediction of the NP structure.

The simplified genome mining flowchart involves: (1) the identification of previously uncharacterized/unknown NP of BGCs within the genomes of sequenced organisms; (2) the sequence analysis of the enzymes encoded in these clusters, including the regulatory elements; and (3) the experimental identification of these NP (Figure 4). 

Genome mining techniques have advanced tremendously in recent years, providing more profound insights into gene expression profiling or an organism’s genetic signature. Since dereplication entails comparing experimental data from new extracts with data from established NP, computational methodologies based on databases are needed to improve the chances of efficiently isolating new molecules [250]. 

Starting from the assumption that elucidation of the genome architecture and structure, in which NP synthetic pathways are encoded, is a central approach to understanding NP chemistry and biology [54], genome annotation (according to Prihoda et al. [29]) represents: 

1. The first step of genome mining and BGC identification. Through an in silico approach, this process leads to the identification and description of the functional elements and function of the predicted gene product in the genome sequence. Pfam is a database of protein families, each represented by Multiple Sequence Alignments (MSA) and Hidden Markov Models (HMMs) (Pfam-A), that is widely used to analyze novel genomes and metagenomes and recently enriched by a set of unannotated, computationally generated MSA called Pfam-B (http://pfam.xfam.org/ (accessed on 22 March 2023)) [251].

The Metashot/prok-quality tool, part of the metashot collection of analysis workflow, is available under a GPL3 license on GitHub. It is a container-enabled Nextflow pipeline for quality assessment and genome dereplication, producing reports that are compliant with the Minimum Information about a Metagenome-Assembled Genome (MIMAG) standard and can run out-of-the-box on any platform that supports Nextflow, Docker, or Singularity, including computing clusters or batch infrastructures in the cloud [252].

BiosyntheticSPAdes is the first automated pipeline for BGC reconstruction, taking advantage of assembly graphs rather than individual contigs, which greatly improves the reconstruction of BGCs from genomic and metagenomics data sets. It is a step towards enabling high-throughput NP discovery by coupling metagenomics and MS data using tools such as NRPquest. BiosyntheticSPAdes allow for the recovery of long BGCs and can be extended to other types of long and highly repetitive genes, such as 16S rRNA genes or insecticide toxins. However, this tool only has predefined options for the most important classes of BGCs (NRPS, PKSs, and their fusions) [253].

2. The second step implies the identification of the BGCs, which is supported by numerous tools that provide linking of genome mining data with known secondary metabolites and by plenty of available reviews that describe those tools and their applications. 

Several bioinformatic tools have been developed or updated, especially in the last two years, such as: antiSMASH (Antibiotics and Secondary Metabolite Analysis Shell), a widely used microbial (and also fungal and plant) GM platform for smBGCs analysis (https://antismash.secondarymetabolites.org/ (accessed on 22 March 2023)) [254], initially released in 2011 [255]; PRISM (http://prism.adapsyn.com (accessed on 22 March 2023)) [256]; BAGEL for visualization of prokaryotic BGCs included in the biosynthesis of RiPPs and (unmodified) bacteriocins (http://bagel4.molgenrug.nl/ (accessed on 22 March 2023)) [257]; and RiPPER specialized for RiPP gene clusters, which is inconvenient for bioinformatic predictions due to the lack of common biosynthetic characteristics (https://hub. docker.com/r/streptomyces/ripdock/ (accessed on 22 March 2023)) [258]. Some of the GM platforms are specialized for targets, such as ARTS (Antibiotics Resistant Target Seeker), which provides efficient GM for antibiotics by rapidly linking housekeeping and known resistance genes to BGC proximity, duplication, and HGT (http://arts.ziemertlab.com (accessed on 22 March 2023)) [259], or TOUCAN, specialized for fungal BGC discovery (http://github.com/bioinfoUQAM/TOUCAN (accessed on 22 March 2023)) [260].

Regarding antiSMASH, one of the most popular genome mining pipelines designed to analyze individual genomes, the recently updated antiSMASH database version 3 (https://antismash-db.secondarymetabolites.org/ (accessed on 22 March 2023)) aims to provide interactive access and cross-genome search functionality based on antiSMASH results for archaeal, bacterial, and fungal genomes [261]. antiSMASH 3 is an upgraded version of this web server tool integrated with the ClusterFinder algorithm, which enables the detection of putative gene clusters of unknown types and also presents a novel dereplication difference of the ClusterBlast module, which identifies similarities of the identified clusters to any of the clusters with known end products [262]. A crucial role in the BGC analysis has also been played by IMG-ABC (Integrated Microbial Genomes Atlas of Biosynthetic Gene Clusters), the database of predicted BGCs combined with experimentally verified BGCs (https://img.jgi.doe.gov/cgi-bin/abc/main.cgi (accessed on 22 March 2023)) [263], and the MIBiG repository (Minimum Information about a Biosynthetic Gene Cluster) as a central reference database for BGCs of known function (https://mibig.secondarymetabolites.org/ (accessed on 22 March 2023)) [193]. Major improvements to the schema, data, and online repository itself, along with extensive manual data curation, are included in MIBiG 2.0 to enhance the annotator quality of the BGC collection and annotations in compliance. Furthermore, it offers user-friendly direct link-outs to chemical structure repositories and new capabilities, including query searches and a statistics page [193].

The vast majority of sequence data in databases was created by advanced high-throughput sequencing, leading to large-scale comparative analysis of homologous BGCs sharing similar domains (termed gene cluster families (GCFs)), the development of BGC/GCF analysis pipelines, and platforms such as BiG-SCAPE (Biosynthetic Gene Similarity Clustering and Prospecting Engine), a software package for grouping GCFs based on the sequence similarity networks of the BGCs. Moreover, the BIG-SCAPE/CORASON workflow enabled the exploration of gene cluster diversity linked to enzyme phylogenies (https://bigscape-corason.secondarymetabolites.org (accessed on 22 March 2023)) [234], BiG-SLICE was designed to cluster massive numbers of BGCs (https://github.com/medema-group/bigslice (accessed on 22 March 2023)) [264], and the BiG-FAM database was devised for performing multi-criterion GCF searches as well as GCF annotation of user-supplied BGCs from antiSMASH output (https://bigfam.bioinformatics.nl (accessed on 8 April 2022)) [265].

3. The third step starts once the BGC information is obtained by the former GM platforms and implies the prediction of the NP structures. Many of the mentioned available tools allow the prediction of NP structures from not only precursor peptides (PP) but also the analysis of (RiPP) BGCs, such as the DeepRiPP three-stage modular platform that combines both genomic and metabolomic information to automate detection of RiPPs and their associated BGCs [266], RiPPMiner for deciphering chemical structures of RiPPs by GM (http://202.54.226.242/~priyesh/rippminer2/new_predictions/index.php (accessed on 22 March 2023)) [267], and RODEO (Rapid ORF Description and Evaluation Online) (http://ripp.rodeo/index.html (accessed on 22 March 2023)) [268]. Gene clusters can also be linked to NP structures using MS data. Strategies based on absence/presence correlations of molecules and gene clusters across strains also allow the connection of MS data to BGCs. 

RiPPquest was the first GM tool to automate both BGC prediction and connection with MS/MS by combining the metabolomic and genome-guided mining tools for the identification of microbial RiPPs [269]. However, this tool was limited to the discovery of lanthipeptides from small databases and could only search for a predefined set of post-translational modifications (PTM). With the aim of solving these limitations, the same team developed the software MetaMiner, which allows matching genomically predicted peptides with their possible modifications to the monomers inferred from MS data. MetaMiner is integrated into GNPS and is also available as part of the NP discovery tool package [270]. Other softwares were released last year, such as CycloNovo for the detection of cyclic peptides (https://github.com/bbehsaz/cyclonovo (accessed on 22 March 2023)) [271] and DeepRiPP, which combines both genomic and metabolomic information to automate the detection of RiPPs and their associated BGCs [53]. The first full-fledged software that automates that process and also introduces a new scoring function was the NPLinker, which also introduces new scoring functions [27] and links genomic and metabolomic data [272].

Pep2Path, freely available at http://pep2path.sourceforge.net/ (accessed on 22 March 2023), paved the way towards high-throughput discovery of novel PNPs by introducing automated MS-guided genome mining for the identification of nonribosomally and ribosomally synthesized bioactive peptides. This tool fully automates the peptidogenomics method through the rapid Bayesian probabilistic matching of MS to their corresponding biosynthetic gene clusters [273].

#### 5.3.3. Chemoinformatics Approaches for Dereplication Using BGCs Diversity

**Figure 5:**
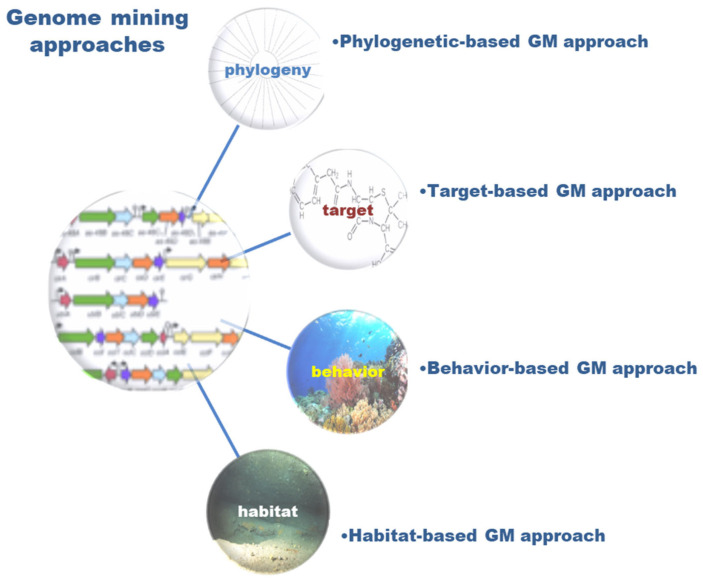
Main strategies in genome mining focused on phylogeny, target, behavior, and habitat approaches.

The combination of interdisciplinary and integrative strategies significantly facilitates and accelerates the process of dereplication. In this way, the different GM strategies can be grouped into the following approaches to mining genomes for NP (Figure 5).

1. The phylogenetic-based GM approach, obtained by comparative genomics, is very useful in predicting the partial or entire molecule structure of a molecule from the gene cluster if another highly similar gene cluster has been linked to a characterized molecule [274]. Gene cluster similarity can be used to find NP with similar functional groups or structures to known compounds, providing a starting point for structural elucidation. When a NP structure is obtained before the annotation of the corresponding genome, genomic data may be used to confirm that the spectrometrically-derived assignments are accurate, or at least compatible with biosynthetic logic [48].

TQMD is available at https://bitbucket.org/phylogeno/tqmd (accessed on 22 March 2023), and it is an optimized tool (comparable with dRep and Assembly-Dereplicator) to dereplicate prokaryotic genomes at higher taxonomic levels (phylum/class) and lower taxonomic levels (species/strains) [275]. 

2. The target-based GM approach is directed to finding specific genes/gene clusters, such as polyketide synthase (PKS) gene clusters [276], putative resistance genes (self-resistance gene mining) [259], or BGC-associated transporter genes that can predict the specialized structure and function of metabolites, such as siderophore activity [277]. The selection of a pathway-specific enzyme as an excellent strategy in the search for BGCs was recently demonstrated by the example of Diels-Alderase-directed genome mining through the analysis of publicly available genomic and metagenomic data of the phyllum Actinobacteria. Using Diels-Alderase (AbyU/AbsU/AbmU) homologues, five complete and 12 partial new abyssomicin BGCs, as well as 23 new potential abyssomicin BGCs, were identified. In addition, this unexpected prevalence of abyssomicin BGC in terrestrial habitats also provided important data on the evolution of abyssomicin BGCs, driven by horizontal gene transfer (HGT), as well as their environmental distribution (mostly in soil and plants) [278]. Indeed, genome and metagenome mining may be used as a preliminary tool in bioprospection, directing the investigations of new NP towards particular taxa and/or unexplored habitats. 

Furthermore, the identification of regulatory elements of the gene cluster, such as promoters and translation initiation signals, may be needed for the purposes of heterologous expression of NP BGCs. Through advanced microbial engineering, synthetic biology seeks to create innovative genetic circuits with practical applications in NP research. 

Thus, Johns et al. [279] used metagenome mining to create a large-scale data set of 169 bacterial and 15 archaeal complete and annotated genomes for constructing a metagenomic regulatory sequence library, which notably expanded the repertoire of prokaryotic regulatory sequences that can be used to construct synthetic circuits, with numerous applications in biotechnology and medicine [280]. Moreover, regulatory sequences with pre-defined host specificities were used to demonstrate programmable species-selective gene expression that produces distinct and diverse output patterns in different hosts. Such species-selective gene circuits (SsGC) with specified host expression profiles provide a framework to engineer synthetic gene circuits with unique cross-species functionality [279].

3. The behavior-based GM approach is inspired by microbial chemical communication, quorum sensing (QS), and wide-spectrum intra- and interspecies interactions, including symbiosis between microbes but also microbes with animals, plants, and fungi [281]. From a NP discovery perspective, symbiotic models can shed light on aspects of the evolution of biosynthetic gene clusters (BGCs) and the manner in which BGCs may contribute to the adaptive fitness of their hosts. It was shown that octocoral-associated species/strains of the genus *Pseudoalteromonas* have amazing genetic potential as a promising source of NP with antimicrobial activity by combining GM, MS/MS molecular networking, and molecular networking with in vitro microbial interactions [282]. However, a significant step forward was made in a recent study that combined pangenome and sequence similarity networks to elucidate the predominant NP that mediates bacterial-nematode-insect interactions within an ecological niche [283]. BGCs from the two Gram-negative genera *Xenorhabdus* and *Photorhabdus* living in mutual symbiosis with entomopathogenic nematodes have been identified. Analysis of 45 strains that represent almost all known strains of these two genera resulted in the identification of 1000 BGCs from 176 families, which provide insight into prevalent bacterial NP that form the functional basis of this tripartite relationship, such as proteasome inhibitors, virulent factors against insects, and insect immunosuppressants. 

4. The habitat-based GM approach provides insights on sampling in different habitats, such as extreme habitats and genome profiling of extremophile organisms [284,285], but also on spatiotemporal metabolic network modeling in complex habitats [286].

Integrative strategies with an interdisciplinary approach successfully unite the different biological and chemical methods in new drug discovery. Trivella and de Felicio postulated in 2018 a tripod for modern drug discovery based on: (1) genome mining; (2) molecular cross-linking based on MS; and (3) growth conditions to induce secondary metabolism as a central strategy for the discovery of new bioactive substances [287]. The importance of an integrative approach that combines genome mining, comparative genomics, and functional genetics/genomics is perhaps best explained by the successful identification of novel biosynthetic gene clusters that produce antimicrobial NP, as confirmed in *Pantoea agglomerans* strain B025670 [274]. Another example of a successful combined approach aiming to facilitate the identification of molecules from complex microbial and plant extracts was a recently established MS-guided genome mining protocol based on GM and MN. In this method, defined as MS-guided genome mining, the main components are previously designated (using MN), and the structurally related new candidates are associated with genome sequence annotations (using GM) [113].

Despite progress in data sharing policies and practices, restrictions are still often placed on the open and unconditional use of various data types, especially genomic data, even after they have received official approval for release in the public domain or in public databases. Such practices are usually against the terms and conditions (i.e., open access mandate) set by the funding agencies, which support research for the benefit of the scientific community and society. Publicly available data should be treated as open data, a shared resource with unrestricted use for analysis, interpretation, and publication, thus promoting the development of new technologies and the advancement of science [288].

## 6. Natural Products Determination of Relative and Absolute Configurations

**Figure 6:**
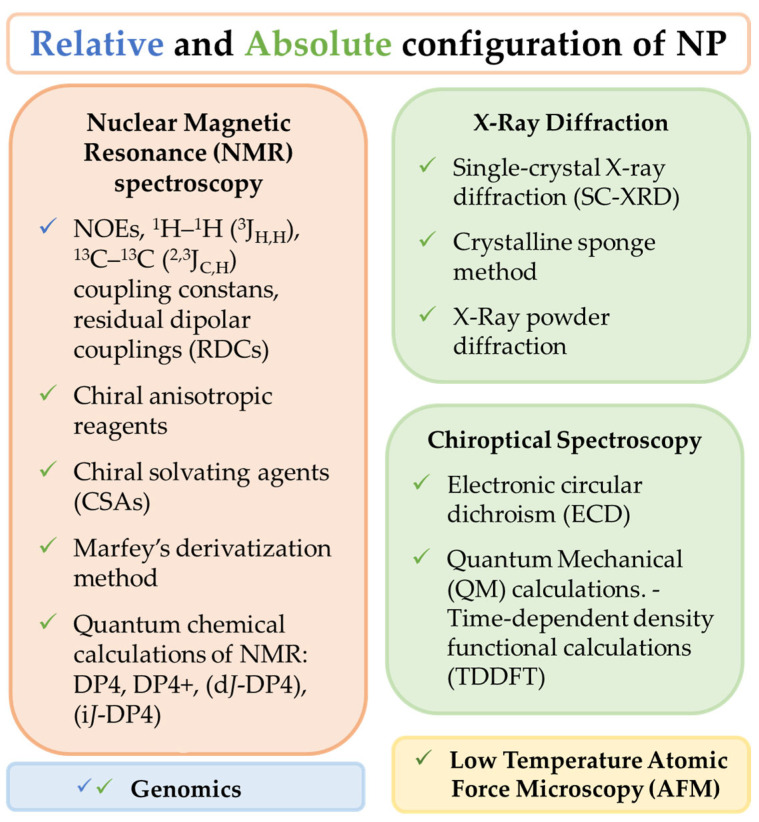
Structure elucidation methods for the determination of Natural Products’ relative and absolute configuration. The methods for the determination of relative configuration are presented in blue check sign (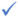) and for absolute configuration determination in green check sign (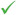).

A key and challenging aspect of NP structure elucidation is the determination of their stereochemistry [30,289]. Knowledge of the molecular shape and spatial features is important to understand the chemical and biological properties of molecules with stereogenic centers. In the pharmaceutical industry, having pure NP or MNP with their 3D structure elucidated is mandatory, as impurities and/or different stereoisomers can have totally adverse effects on human health, such as in the case of Thalidomide^®^. Numerous strategies have been developed to overcome limitations, including the scarce amount of sample availability, the presence of stereogenic centers, multiple chiral quaternary atoms, or the chirality of flexible systems [290,291]. Depending on the specific physical and chemical characteristics of NP, their stereochemical features can be studied by X-ray diffraction, chiroptical methods, chemical derivatization, NMR-based methods, computational NMR methods, and genomics (Figure 6).

### 6.1. X-ray Diffraction 

Single-crystal X-ray diffraction (SC-XRD) is a valuable method for the structural elucidation of NP. It provides information on molecules at the atomic level and can be used to determine their absolute configuration. The main limitation of this technique is the requirement of high-quality single crystals for the analysis, which may be complicated to obtain. Many NP are not crystalline, and, usually, the scarce amounts of substance isolated may interfere with the quality of crystals for X-ray diffraction. 

In recent years, two methods have been described that allow X-ray diffraction without the need for crystalline compounds.

The first is the crystalline sponge method, based on the use of a crystalline molecular flask (CMF), which, in its solid state, possesses high tolerance to structural deformation without loss of crystallinity. These materials can absorb the target molecule and arrange it in a highly organized manner, allowing X-ray analysis of the sample. Therefore, the X-ray technique can be extended to non-crystalline NP and to NP that have been isolated in very small amounts since the crystallographic analysis can be performed at the ng to µg scale. This method was described for the first time in 2013 [292], and since then, it has proven to be suitable for the determination of the absolute configuration of small molecules, including those containing chiral quaternary carbons [293]. It has been used to determine the absolute configuration of NP belonging to a variety of skeletons, such as elatenyne, which presents a pseudo-meso core structure [294], or the sesquiterpenes cycloelatanenes A and B, which are epimers and possess five chiral quaternary atoms [295]. The chemical properties of the crystalline molecular flask (CMF) are essential to the application of this method, as the Metal−Organic Framework (MOF) used by Fujita and co-workers decomposes in contact with Lewis basic or protic substituents [292]. Therefore, great research efforts are currently being carried out focused on the development of new CMFs to optimize the method and expand the array of solvents and compounds that can be used [296,297,298].

The second is the X-ray Powder Diffraction method, which allows NP structure determination from powder diffraction data (SDPD). Although X-ray powder diffraction can only differentiate diastereomers, this method has been used to establish the absolute configuration of acidic or basic compounds by the formation of salts with chiral counter ions. The crystal structure of the salts is analyzed by X-ray powder diffraction, and the absolute configuration can be deduced from the known chirality of the counter ion [299]. So far, this methodology has proven useful with the acids (*R*)-flurbiprofen and (*S*)-flurbiprofen, using quinine and (*R*)-2-phenylpropylamine as counter ions. In addition, the absolute configuration of the basic compounds aminoglutethimide and lamivudine was determined using (*R*)-camphor-10-sulfonic acid as a counter ion. The preparation of the crystalline salts has been reported on the mg to µg scale. Thus, this methodology could be suitable for the scarce amounts of NP commonly isolated from natural sources. One limitation of this method is the need for compounds that can form salts with suitable counter ions and the need for good-quality crystals of the obtained salts.

### 6.2. Chiroptical Spectroscopy

The interaction of a chiral NP with circularly polarized light determines its absolute configuration. Chiroptical methods are non-destructive and do not require crystallization or the use of chiral auxiliaries. Currently, there are several chiroptical methods based on circular dichroism (CD) used for the determination of the absolute configuration of NP.

Electronic circular dichroism (ECD) is defined as the differential absorption of circularly polarized radiation in the UV-Vis region of the electromagnetic spectrum. Therefore, it deals with CDs that originated from molecular electronic transitions. ECD has been extensively used for the assignment of AC and conformational studies of NP. Its main advantage is its high sensitivity, since a good spectrum can be obtained on the sub μg scale. Even though originally this method could not be used for NP lacking an UV/Vis active chromophore and was not entirely accurate for flexible molecules, the use of chiral probes has expanded its use. Chiroptical probes are achiral moieties that can be attached to a chiral compound. Ideally, these probes should introduce rigidity and chromophores that enhance the chiroptical response in the ECD spectrum. For example, biphenyl chiroptical probes have been recently used for the determination of the absolute configuration of colletochlorin A and agropyrenol, two flexible phytotoxins isolated from the fungal pathogens *Colletotrichum higginsianum* and *Ascochyta agropyrina*, respectively [300,301,302].

The interpretation of the spectrum obtained by ECD can be done by comparison with a reference spectrum; by correlation with similar compounds; using empirical rules; or using the exciton chirality approach. All these approaches are limited because they focus on a few transitions of a specific chromophore and depend on a collection of experimental data.

During the last decades, the development of computer technology and Quantum Mechanical (QM) calculations has had a tremendous impact on the use of chiroptical analysis for AC determination. At present, there are numerous examples of the use of QM calculations for the determination of the absolute configuration of complex natural products [303,304].

Time-dependent density functional calculations (TDDFT) allow, with relatively low computational calculations, a reasonable accuracy in the prediction of excitation energies and rotational strengths, whereas coupled cluster calculations are limited to small molecules due to their higher computational calculations. It is important to point out that flexible molecules may have several conformers that contribute to the optical properties of a chiral compound and must be considered. Therefore, it is very important to define the possible conformers before conducting quantum-mechanical calculations. 

### 6.3. Low Temperature Atomic Force Microscopy (AFM)

In 2018, Schreiner et al. assigned the absolute configuration of the two tetramantane enantiomers by direct visual inspection using low-temperature atomic force microscopy (AFM) with a CO-functionalized tip [305]. The experimental results were supported by computational studies. 

The absolute configuration was assigned by differentiation of the two enantiomers on a Cu (III) surface and by visualization of characteristic hydrogens. The molecules were deposited onto the Cu surface at a temperature of −258.15 °C; therefore, this procedure could be suitable for volatile compounds. In addition, there is no need for chromophores for particular atoms or functional groups. This work indicates that microscopic techniques could become standard tools for absolute configuration (AC) determination in the future [306].

### 6.4. Relative Configuration by NMR

NMR spectroscopy is the first-choice method to study the relative configuration of NP. It is a non-destructive technique that allows, without chemical transformation, to determine most of the relative configurations of complex NP with several chiral atoms.

The assignment of stable conformations and relative configurations of a chiral NP without any chemical transformation can be deduced from the study of nuclear Overhauser effects (NOEs), two- and three-bond ^1^H–^1^H (*^3^J_H,H_*), ^13^C–^1^H (*^2,3^J_C,H_*) coupling constants, and also from the study of residual dipolar couplings (RDCs). The interpretation of homonuclear coupling constants (*^3^J_H,H_*) and NOEs should be enough to establish the relative configuration of cyclic or rigid NP that have a limited number of conformers, and this method has been widely used.

Linear or cyclic flexible NP may present more difficulties for the assessment of stable conformers and the assignment of relative configuration. In 1999, Murata et al. described a method to assign the relative configuration of stereogenic methine carbons based on the analysis of ^1^H–^1^H *^3^J_H,H_*, ^13^C–^1^H *^2,3^J_C,H_* coupling constants, and nuclear Overhauser effect (NOE or ROE) interactions [307]. This method, also called *J*-based configurational analysis, can be applied to acyclic compounds and to larger, flexible macrocyclic structures. For flexible systems, the relative conformation of adjacent stereogenic centers can be represented by six staggered rotamers. For each configuration, the chiral methine protons have an anti-orientation in one rotamer and gauche-type orientations in the other two rotamers. If this system adopts one main conformer (>85% of the total), analysis of the homonuclear and heteronuclear coupling constants of the methine protons can be useful for the determination of the relative configuration of those carbons. This method can also be applied to 1,3-methines and even to 1,4-methines if the methylene protons are well resolved. This method has been extensively used for the establishment of the relative configuration of many NP [308,309]. This method relies on the ability to determine ^1^H-^1^H and ^1^H-^13^C coupling constants. Vicinal ^1^H–^1^H couplings can often be measured from ^1^H NMR spectra, and for overlapped resonances, selective pulse sequences such as 1D TOCSY may eliminate overlapping signals and allow direct measurement of ^1^H–^1^H coupling constants. On the other hand, the measurement of long-range ^1^H–^13^C couplings has been very challenging due to the low naturally abundant ^13^C nucleus and the difficulties in accurately measuring small ^1^H-^13^C coupling constants (<2.3 Hz). In the last few years, a variety of 2D NMR experiments have been described for determining *^n^J_C,H_* coupling constants, for example, HECADE; HSQC-TOCSY; *J*-HMBC; EXSIDE; selEXSIDE; S^3^-HMBC hetero; HMBC-IPAP; or HSQMBC [310]. Still, there is not a general method that can overcome all the limitations associated with these coupling constants. For example, HMQC-TOCSY and HSQC-TOCSY only work for protonated carbons, and HMBC and HSQMBC experiments deliver complex multiplets due to simultaneous *J_H,H_* phase modulation, which makes the analysis of coupling constants very complicated [311]. Therefore, this is an area of active research, with new experiments being added periodically, such as the LR-selHSQMBC experiment, which allows for the observation of weak heteronuclear correlations that can be potentially missing in standard HMBC/HSQMBC experiments [312].

The relative configuration of NP that present specific features or functional groups can be deduced from the observation of ^1^H and ^13^C NMR chemical shifts, and through the years of investigation and observation of chemical shifts of different types of compounds, several NMR empirical rules have been established: The relative configurations of aryl-glycerols can be determined by the ^1^H NMR chemical shift differences of the diastereotopic methylene protons [313]. In addition, the *^3^J_H,H_* values allow the assignment of threo and erythro configurations of polyacetylene glycosides [314]; the relative configuration of 1,3-methyl-branched carbon chains can be determined by study of Δδ of relevant methylene protons [315]; and the relative configuration of fatty acid butanolides isolated from an octocoral of the genus *Pterogorgia* was established by the study of the ^13^C NMR chemical shifts of the carbons on the 3-alkyl-4-hydroxy-5-methyl-2(5H)-dihydrofuranone ring, a γ-lactone motif ubiquitous in many bioactive natural products [316]. The geometry of vicinal vinyl dihalides can be established by the observation of the ^1^H and ^13^C chemical shifts of C-1 [317].

### 6.5. Absolute Configuration by NMR

NMR spectroscopy can be useful to determine the absolute configuration of NP by derivatization with chiral anisotropic reagents or using chiral solvating agents (CSA) through non-covalent interactions associated with the chiral NP of study.

#### 6.5.1. Derivatization with Chiral Anisotropic Reagents

This methodology has been extensively used for the study of NP containing secondary alcohols, α-substituted primary amines, and α-substituted carboxylic acids.

Two enantiomers of a chiral anisotropic reagent are used to derivatize, separately, the NP under study to obtain two epimers whose chemical shifts around the stereogenic center will be affected by the aryl group of the anisotropic substituent. Protons that reside where the magnetic lines of force for the induced magnetic field oppose the applied field experience a shielding effect and are shifted upfield, while protons situated where the induced magnetic field complements the applied field are deshielded and shifted downfield. Conformational studies and differences in those chemical shifts allow the establishment of the absolute configuration of the stereogenic center.

In 1973, Mosher et al. described the empirical correlation between the configuration of a chiral alcohol and the NMR chemical shifts of the diastereomeric products that result from reactions with specific chiral esterification reagents containing an aryl substituent [318,319]. Later, the correlation between ^1^H NMR chemical shifts of the ester derivatives of the R- and S-α-methoxy-α-trifluoromethylphenylacetic acids (MTPAs) was elaborated into the advanced Mosher’s method [320,321]. Therefore, this method has been extensively applied to determine the absolute configuration of NP containing secondary hydroxyl groups by derivatization and ^1^H NMR analysis. This method is also applicable to stereogenic methine carbons bearing a primary amine group. There are certain NP where the method cannot be reliable, for example, NP in which steric factors produce conformations that deviate from the proposed model or heavy signal overlapping of the protons around the stereogenic center of study. 

Besides MTPA, there are other chiral anisotropic agents that can be applied to the modified Mosher’s method, such as methoxyphenylacetic acid (MPA), 9-anthrylmethoxyacetic acid (9-AMA), and phenylglycine methyl ester (PGME). PGME can be applied to elucidate the configuration of methine carbons that are α-positioned to carboxylic acids [322,323]. The use of methoxyphenylacetic acid (MPA) as a chiral auxiliary improves the Δδ acquisition of ^1^H NMR at low temperatures or by adding barium salts to the NMR tube. 9-AMA allows the use of this methodology for the determination of the absolute configuration of NP by derivatization of primary alcohols, and the use of MPA or 9-MPA in combination with low temperatures or the use of barium salts allows the determination of secondary alcohols or primary amines from just one single derivative [324]. Moreover, the methodology of preparation of the derivatives has evolved to reduce or eliminate steps of purification and simplify the experimental process. For instance, the use of auxiliary reagents attached to polymeric supports has allowed the preparation process to be carried out in the NMR tubing [325,326].

More recently, this methodology has been extended to the determination of the absolute configuration of polyfunctional NP possessing two or more close chiral atoms. For these NP, the analysis of the Δδ_RS_ takes into consideration the crossed effects between auxiliaries of the derivatives as well as conformational studies of each derivative to predict the shielding signs of the Δδ_RS_ of the protons of the molecule [327]. In addition, besides ^1^H NMR experiments, ^13^C NMR of the derivatives has been analyzed to open this methodology to substrates without protons directly bonded to an asymmetrical carbon atom [328].

#### 6.5.2. Chiral Solvating Agents (CSA)

The non-covalent interactions between CSA and the compound under study can be used to assign the absolute configuration of NP. Usually, this methodology implies a mixture of the CSA and the NP in the NMR tube.

Separately, two enantiomers of a CSA are used to form stable diastereomeric complexes with the chiral compound. Usually, a CSA possesses a strong anisotropic group that should produce selective shielding effects around the stereogenic center of study. The study of the stable conformation of those resulting CSA-compound complexes and the Δδ of selected atoms allows the establishment of the absolute configuration of a specific stereocenter.

There are numerous examples of CSA used to determine the absolute configuration of NP. For example, 2,2,2-trifluoro-1-(9-anthryl)ethanol (TFAE) was initially used to establish the absolute configuration of the γ-methyl butenolide moiety of the NP isolated from annonaceous acetogenins [329]. Later, this methodology has been used to determine the absolute configuration of γ-methyl butenolide diterpenoids of sponges [330], furanocembranolides of octocorals [331], and even to establish the absolute configuration of sesquiterpenes isolated from red algae possessing a γ-butenolide or δ-lactone moieties [332].

The use of a particular CSA is restricted to compounds containing specific functional groups; therefore, there is a continuous search for new CSAs that can be useful to determine the absolute configuration of compounds containing diverse functional groups. More recently, new protocols have been published to determine the absolute configuration of compounds that contain acids, esters, hydroxy acids, and amino acids that interact with CSA [333,334,335,336].

#### 6.5.3. Absolute Configuration of Amino Acids by Marfey’s Derivatization Method

The resolution of enantiomers can be achieved by indirect approaches: each enantiomer reacts with a chiral derivatizing reagent (CDR) to produce a pair of diastereomers that can be easily separated by chromatography without the requirement of chiral support. For this approach to be useful, there are certain conditions: the enantiomer molecule and the chiral derivatizing reagent (CDR) must possess compatible and easily derivatizable functional groups; the reaction should be rapid; and the CDR must possess a chromophore to enhance the chromatographic detection.

In 1984, Marfey published a method for the determination of L- and D-amino acids by chiral derivatization with 1-fluoro-2,4-dinitrophenyl-5-L-alanine amide (L-FDAA) [337]. Briefly, L-FDAA contains a reactive fluorine atom that is used for the reaction with a mixture of L- and D-amino acids, and the resulting diastereoisomers can be separated and analyzed by reverse-phase HPLC, where very distinct retention times are obtained for both diastereoisomers. This method has become very popular for the establishment of the absolute configuration of many natural metabolites containing amino acids, especially peptides [338,339,340]. The first step of Marfey’s method is the acid hydrolysis of the NP to obtain the amino acid residues. Then, the hydrolysate is derivatized under alkaline conditions with L-FDAA to obtain a mixture of L-FDAA derivatives of the constitutive amino acids of the peptide that can additionally be separated and analyzed by HPLC. The retention time of each derivatized amino acid can be compared to that of the derivatized L- and D-amino acid standards. Marfey’s derivatives of D- and L-amino acids can be identified by co-injection of standard derivatized D- and L-amino acids.

Since the publication of the original method, there have been some improvements: new chiral reagents have been prepared by the reaction of 1,5-difluoro-2,4-dinitro benzene (DFDNB) with Val–NH_2_, Phe–NH_2_, and Pro–NH_2_, amino acids with carboxyl groups or amino acid amides, among others [341], and the “advanced Marfey’s method”, which combines the Marfey’s method with FAB and ESI/MS [342,343]. 

One of the drawbacks of Marfey’s method is the analysis of amino acids that possess a second stereocenter at Cβ, such as isoleucine (Ile), due to the lack of chromatographic resolution of all possible stereoisomers, l-Ile, l-*allo*-Ile, and d-Ile, d*-allo*-Ile. The “C_3_ Marfey’s method”, which uses a C_3_ stationary phase instead of the most common C_18_ for HPLC analysis at a temperature of 50 °C and a ternary gradient, has proven to achieve the separation of these epimers [344]. More recently, another approach using tandem HPLC-SPE-NMR based on the differentiated NMR data of these epimers have been described [345]. 

#### 6.5.4. Quantum Chemical Calculations of NMR Parameters

In the last decades, computational chemistry methods using quantum mechanics and molecular mechanics theories combined with statistical approaches have evolved rapidly [33,304]. Consequently, theoretical calculation models for the determination of NMR parameters have allowed comparison between experimental and computational data, becoming a powerful tool to aid in the structural and stereochemical determination of natural molecules. These methods have been successfully used to characterize and revise the structures of natural and synthetic products [33].

In general terms, the procedure to determine the most likely structure among several stereoisomers involves a conformational search to explore possible conformers of candidate molecules, followed by geometry optimization, then calculation of NMR properties, molecular energy calculations, Boltzmann averaging, and finally comparison of the calculated values with those obtained from experiments [32]. In this last step, it is crucial to choose an appropriate statistical method. Among them, it is worth mentioning the CP3 [346], DP4 [347], DP4+ [348], and *J*-DP4 [349] probability methods. 

The CP3 and DP4 methods were introduced by Goodman and coworkers. The CP3 parameter improved results obtained by other statistical descriptors such as R^2^, mean absolute error (MAE), or corrected mean absolute error (CMAE). Thus, for a pair of diastereoisomers, NMR chemical shift calculation, combined with analysis using CP3, was an effective way to assign two experimental spectra to two possible structures [346]. However, the method had limited application in NP research. This problem was solved with the DP4 method, designed to identify stereochemistry among multiple candidate stereostructures with one single set of experimental NMR chemical shifts available [347]. The DP4 has been extensively used in the structure elucidation of many complex NP, such as the complete reassignment of the alkaloid echivulgarine, obtained from pollen of *Echium vulgare* [350]. Other examples are the determination of the unsolved absolute stereochemistry of cyclocinamide A, a 14-membered cyclic peptide with four unrelated stereocenters for application of the DP4 protocol to a simplified synthetic peptide core [351], or the stereochemical determination of marilzafurollenes A–D and 12-acetoxy-marilzafurenyne, five halogenated C_15_ tetrahydrofuranyl-acetogenins isolated from *Laurencia marilzae*. In this case, despite the methodology allowed to connect remote stereocenters, the presence of halogens, frequent in marine metabolites, interfered with reliable calculations [352] and lacked accuracy in flexible molecules [353]. DP4+ was introduced in late 2015 by Sarotti and coworkers [348] as an improvement of DP4, with the inclusion of a geometrical optimization step and the use of a higher level of theory for NMR calculations. The absolute configuration of the novel estrogenic α-pyrone, arthrifuranone A, was established by combining the Mosher’s method and gauge-including atomic orbital NMR chemical shift calculations, followed by DP4+ analysis [354]. Despite DP4+’s better performance, it requires a higher computational cost, as do other improved methods such as DP4.2 [355] and DiCE (diastereomeric in silico chiral elucidation) [356]. With the aim of obtaining better results than the original DP4 method and reducing the associated computational costs, the *J*-DP4 method was developed by incorporating vicinal coupling constants (*^3^J_H,H_*) into the analysis in three possible ways: direct (d*J*-DP4), indirect (i*J*-DP4), and d*J*/i*J*-DP4. [349] These potent and sophisticated tools remain rapidly evolving, becoming progressively determinant in the structure elucidation of large and flexible molecules [357]. 

### 6.6. Relative and Absolute Configuration Aided by Genomics

As we showed in Section 5.3, the biosynthetic information found in the genome of organisms can be used to predict metabolite molecular frameworks. Furthermore, knowledge of the stereospecificity of biosynthetic enzymes can be used to predict the configuration of NP. This approach can be especially useful for the assignment of the full absolute configuration of complex NP with multiple stereogenic centers, which would require a combination of approaches including X-ray diffraction, 2D NMR analysis, the preparation of chiral derivatives, partial degradation, or analogue and asymmetric synthesis.

A good example of structural complexity is macrolides, microbial metabolites characterized by a large lactone ring to which can be attached one or more sugars and multiple hydroxyl or alkyl groups. These NP present a high number of stereogenic centers and high flexibility, which, together with the presence of isolated stereocenters, complicate the assignment of their absolute configurations. Macrolides are produced by type I polyketide synthases, and many of the enzymes that mediate their synthesis are highly stereospecific; therefore, it is feasible to use the knowledge of the enzyme stereospecificity to predict the absolute configuration of macrolides.

In recent years, the absolute configuration of several macrolides has been described by the analysis of genomics and a combination of NMR data analysis and/or quantum mechanical calculations. For example, the absolute configurations of polyketides niphimycins C−E, isolated from a marine-derived *Streptomyces* sp., have been proposed from the analysis of the ketoreductase and enoylreductase domains for hydroxy- and methyl-bearing stereocenters [358]. In addition, in 2018, the full absolute stereostructure of neaumycin B was proposed [359]. More recently, the absolute configurations of the formicolides were proposed based on the application of ketoreductase amino acid sequence analysis and quantum mechanical calculations [360].

## 7. Computer Assisted Structure Elucidation and Related NP Databases 

**Figure 7:**
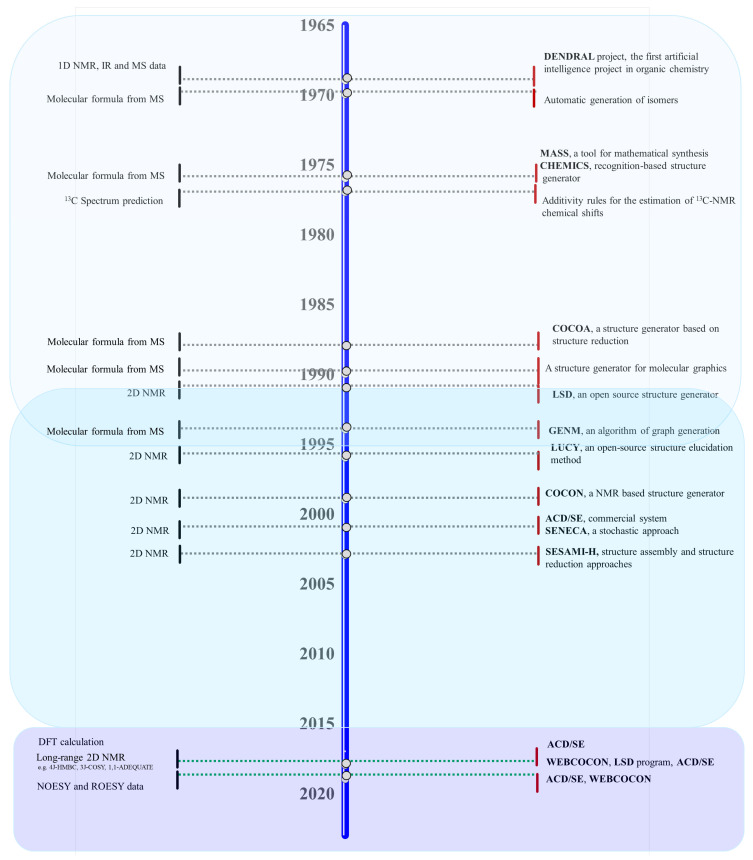
Timeline illustrating the major advances in CASE systems, period 1969–2023. The boxes represent the three phases highlighted in the development of the CASE. Phase I between 1969 and 1994 is represented in light blue, and Phase II between 1991 and 2016 is in blue. Phase III between 2016 and 2023 is in purple.

Computer assisted structure elucidation (CASE) has been a well-established system in the chemical community for more than 50 years. The elucidation of the structure of NP is, by its nature, a very complex process in which any available information that can be used to elucidate the structure of an unknown compound cannot be ignored. Despite great advances in spectroscopic techniques, there have been in recent years a surprisingly high number of cases in which a previously reported NP structure was later shown to be incorrect. Therefore, CASE systems have a high relevance by integrating all existing computational methods, for example, structure generation by structure assembly [361,362,363,364] and reduction [365], stochastic structure generators [366], combinatorial structure generation with restraints [367,368], convergent structure generation [369,370], fuzzy structure generation [371], chemical graph generators [42], logic engines [372], combinatorial brute force [373,374,375,376], databases of ^13^C NMR chemical shifts and fragments [377,378], genetic algorithms [379,380], simulated annealing [381], evolutionary algorithms [382], expert systems [44,383], and expert systems with Density Functional Theory (DFT) [43,44,45]. In Figure 7, the main achievements of CASE systems are highlighted [39,40,41,43,384,385].

The evolution of the CASE systems in the past fifty years clearly highlights three approaches, shown in Figure 7. Phase I between 1969 and 1994 is represented in light blue, Phase II between 1991 and 2016 in blue, and Phase III between 2016 and 2023 in purple. The CASE system was built considering the following data: 1D NMR/IR/MS, 2D NMR, and 2D NMR/DFT/NOESY/ROESY estimation for phases I, II, and III, respectively. 

**Figure 8:**
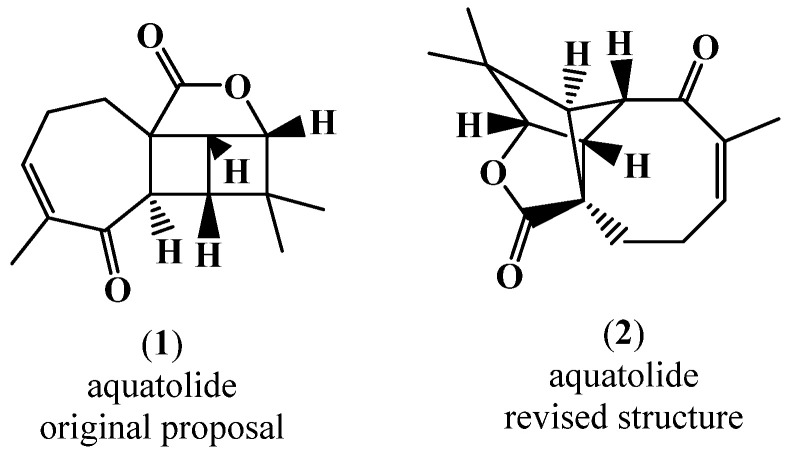
Originally proposed structure of aquatolide (**1**) and revised aquatolide structure (**2**).

In general, CASE systems produce a set of possible structures that satisfy the experimental spectroscopic data and the CASE knowledge. Depending on the number of restrictions imposed by the CASE system, the output file size can vary widely, from a small number of structures to hundreds of thousands. To select the most likely structure, CASE systems use the prediction of 1D NMR chemical shifts (e.g., ^13^C, ^1^H, ^15^N, ^19^F, and ^31^P) by empirical methods. To hierarchize the structures, a comparison between predicted and experimental ^13^C chemical shifts is performed by CASE systems. Only recently has the DFT-based quantum mechanics (QM) approach has achieved greater accuracy when compared to empirical methods. For example, Lodewyk et al. [386] reported the revision of the structure of aquatolide (**1**), a humulane-derived sesquiterpenoid lactone, based on DFT calculations of ^13^C chemical shifts and subsequently confirmed by X-ray crystallography as having the revised structure (**2**) (Figure 8). 

In this study [386], DFT-based ^13^C chemical shift calculations clearly showed the validity of structure (**2**), the revised one, the corrected mean absolute deviation (CMAD) (**2**) = 1.37 ppm, and CMAD (**1**) = 7.23 ppm. Despite all the merits of DFT-based chemical shift prediction, current empirical methods remain indispensable for efficiently generating and selecting the most likely structure or structures in large CASE output files, mainly due to their high speed and reasonable accuracy [39]. The power of CASE is that it generates all possible structures and then performs a fast selection of the most likely structures based on efficient chemical shift calculations. The critical function of CASE is its capability to generate structures that cannot be performed by DFT calculations. While CASE programs’ chemical shift predictions are generally reasonable, their accuracy is highly dependent on the structures being analyzed and can vary significantly between structures. Thus, the accuracy of CASE chemical shift predictions of aquatolide was as low as 6 ppm, which justified the application of DFT computations for the shortlist of CASE-generated structures for aquatolite [44]. The revision of the aquatolide structure is a perfect example of the superior accuracy of chemical shift predictions by DFT calculations as well as the synergistic power of combining CASE and DFT methods. The original revision of the aquatolide structure took more than a year [386], while the CASE-DFT revision took just a few hours [44]. Therefore, in the CASE system such as ACD/SE, the use of DFT calculations of ^13^C and ^1^H chemical shifts was proposed in a final phase for the short list of structures generated by their program to support a more conclusive choice about the most probable structure and to determine relative stereochemistry, if needed [39,44,45]. 

Very promising approaches using machine learning and deep learning methodologies were also explored to quickly and accurately predict NMR chemical shifts using large databases of high diversity [387,388]. Jonas et al. [387] reported the use of deep neural networks for predicting NMR shifts, achieving a precision of 1.43 ppm mol MAE for ^13^C and 0.28 ppm mol MAE for ^1^H shifts using the data available in nmrshiftdb2 (https://nmrshiftdb.nmr.uni-koeln.de/ (accessed on 18 January 2023)) as input data. Even better performance is achieved with the approach developed by Kwon et al. [388] using an improved method based on enhanced molecular graph representation and a message passing neural network (MPNN) for ^13^C and ^1^H NMR chemical shift prediction, achieving MAE values of 1.36 and 0.22 ppm, respectively. Although CASE remains a challenge [4,40,41,43,389,390], there is a clear synergistic interaction between new NMR techniques, computational chemistry methods, and the evolution of CASE systems. In this way, the new CASE protocols incorporate advances in experimental and theoretical techniques such as powerful new correlation experiments e.g., LR-HSQMBC (Long-Range Heteronuclear Single-Quantum Multiple Bond Correlation), HSQMBC–TOCSY (Heteronuclear Single-Quantum Multiple Bond Correlation–Total Correlation Spectroscopy), new and orthogonal techniques e.g., RDC (Residual Dipolar Couplings) data, RCSA (Residual Chemical Shift Anisotropy) data, DFT prediction of chemical shifts followed by DP4 probabilities calculation using vibrational effects, and deep learning, a new powerful approach to computational science based on neural networks.

## 8. Chemoinformatics Tools to Facilitate Drug-Lead Discovery 

**Figure 9:**
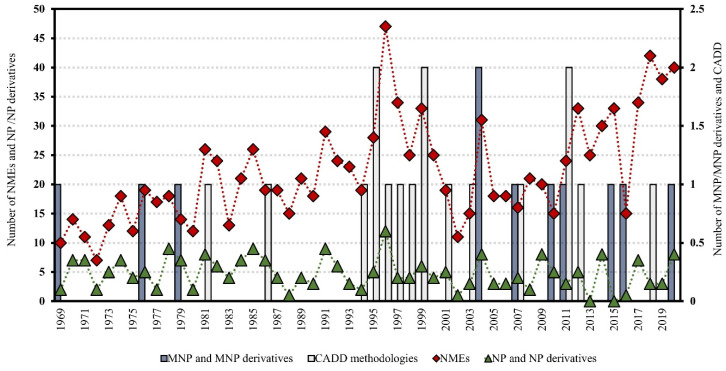
Novel FDA approvals during 1969–2020, where NMEs are all the approvals except biologics license applications; NP and NP-derivatives; and MNP and MNP-derivatives; CADD methodologies refer to drug approvals that were developed using CADD. MNP are approved drugs by the most representative approving agencies, such as the U.S. FDA, the European Medicines Agency (EMA), the Japanese Ministry of Health, and Australia’s Therapeutic Goods Administration. Data are from Drugs@FDA and the literature [250,391,392,393].

Statistics concerning novel drug approvals by the Food and Drug Administration (FDA) during 1969–2020 showed a very diverse behavior since the peak in 1996, with 47 new molecular entities (NMEs)/year, and the minimum (after 1996) of 11 NMEs/year in 2002. Figure 9 updates the global number of new FDA approvals with the number of NP and NP derivative approvals until 2020, highlighting the contribution of MNP and computer-aided drug design (CADD) methodologies that were reported in the review by Pereira and Aires-de-Sousa [250]. 

During the COVID-19 pandemic, the FDA approved 40 NMEs in 2020; this is the third highest number of approved compounds obtained since 1969, falling only slightly short of the value obtained of 42 in 2018 and the value of 47 in 1996 (Figure 9). In the last decade, 2011–2020, there was a clear upward trend in NMEs/year, with a 10-year average of 31.4 NMEs when compared to the previous decade, 2001–2010, with a 10-year average of 18.4 NMEs. In the case of NP and NP-derivatives, there was a constant behavior over time, with a 10-year average of 4.2 and 3.8 for both the decades 2001–2010 and 2011–2020, respectively (Figure 9). Curiously, the high point for NP and NP derivatives was in 1996 (with 12 approved drugs), and the 1990s decade was also the most successful for CADD-driven drugs, with eight approved drugs. However, more than half of the total approvals of MNP and MNP derivatives occurred in the 21st century (eight out of eleven approved drugs) (Figure 9). 

New approaches are needed to overcome the perceived disadvantages of MNP when compared with synthetic drugs, such as the difficulty in access and supply that made the investigation of MNP only began in the 1980s [3,250,394]. Marine-based drug development is a time-consuming and costly endeavor that takes between 17 years (e.g., trabectedin, Yondelis^®^) and 24 years (e.g., halichondrin, Halaven^®^; dolastatin, ADCetris^®^), with an average of 23 years from the MNP discovery to marketing [3]. To overcome these difficulties, CADD approaches can be used to guide decisions concerning the in vivo and in vitro testing of isolated NP and extracts, to assist in the design of bioactive NP derivatives, and to virtually screen databases of known or proposed NP. Thus, it is important to understand where in chemical structural space biologically relevant compounds are found and the relationship between these two spaces (i.e., chemical-biological). 

The regions of the chemical space surrounding NP are recognized as promising for the development of new drug leads, according to a comprehensive analysis covering the period between 1981 and September 2019. The NP scaffolds, which include unaltered NP, NP derivatives, and NP mimetics and/or contain an NP pharmacophore, represent 45% of all approved small-molecule drugs [395]. A statistical analysis of the structural classification of NP performed by Waldmann and co-workers [396] showed that more than half of all NP have the right size (i.e., a van der Waals volume between 300 and 800 Å^3^) to serve as a starting point from hit to lead discovery. Likewise, in a different subset of PubChem, Pereira et al. [397] have also reported a correlation between active compounds and three- or four-ring compounds with a van der Waals volume between 300 and 800 Å^3^. A NP-likeness score to measure the similarity between a molecule and the structural space covered by NP was developed by Ertl et al. [398] and incorporated in SENECA, an open-source CASE platform [399]. 

**Figure 10:**
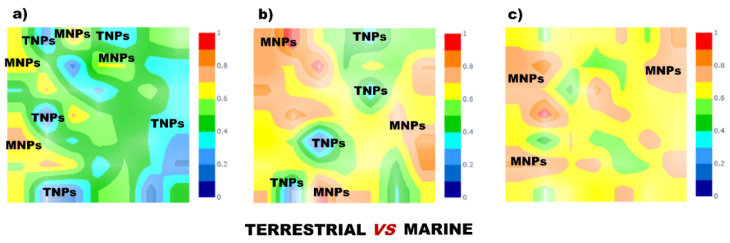
GTM terrestrial and marine origin of NP landscape for: (**a**) the external test set; (**b**) the StreptomeDB 2.0 database; and (**c**) the Pye data set [401]. Dark blue, or 0, represents the TNP class, and red, or 1, represents the MNP class.

More recently, two complementary works were reported by Shang et al. [400] that analyzed the differences between terrestrial natural products (TNP) and MNP using chemoinformatics methods and Pereira et al. [401], which performed machine learning (ML) modeling to predict the terrestrial and marine origins of NP. Both studies reported a trend for MNP to have more halogens (especially bromine) and fewer oxygen-containing groups than TNP [400,401]. However, different conclusions were obtained about the size of the rings in these two studies [400,401]. The first study [400] reported that larger rings, especially 8- to 10-membered rings, were generally present in MNP, unlike the second study [401] that reported that 5-membered rings were more relevant in the discrimination of the MNP. A clear separation between the chemical space represented by MNP when compared to TNP when exploring ML techniques was observed [401]. A Generative Topographic Mapping (GTM) for chemical data visualization was also developed [401] in order to map the terrestrial and marine origin of the NP landscape for the external test set (a data set not used to build the model, comprising 3236 MNP and 3258 TNP) for the StreptomeDB 2.0 database (2877 unique microbial NP produced by the genus *Streptomyces*, an actinobacterium) [402] when comparing with the Pye data set (5486 unique microbial and MNP) [58,403,404,405,406] (Figure 10).

**Figure 11:**
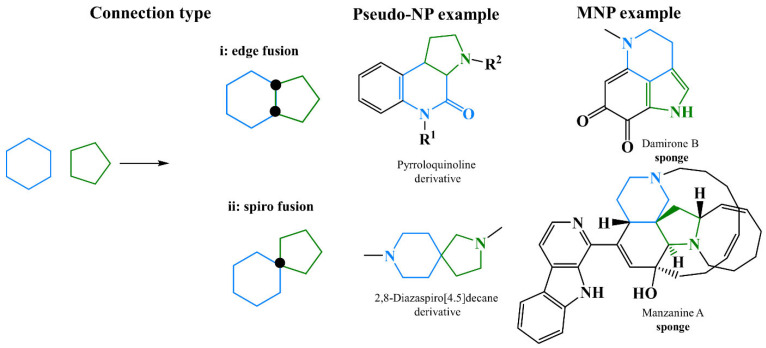
Demonstration of NP fragment connectivity such as (i) edge fusion and (ii) spiro fusion to guide the synthesis and design of *pseudo-NP*. These connectivity patterns are also found in MNP, and representative examples are shown. Black dots denote connectivity points. Individual fragments were indicated in blue or green [404].

Interestingly, an overlap between the chemical space of microbial NP and MNP can be seen in Figure 10, but also taking into account the predictions with ML models carried out in Pereira’s work, which predict MNP at more than 64% for the StreptomeDB 2.0 database and for the Pye data set. There are undoubtedly three key criteria for designing compound libraries to model protein function: diversity, drug-likeness, and biological relevance. The unique structural features of NP were explored using various approaches to making NP-derived fragment databases for fragment-based drug discovery. Generating and making these fragments publicly available were also explored. To identify structurally diverse compounds that share the same biological activity space, the concept of scaffold hopping was developed in 1999 [407]. Initial application of virtual screening of scaffold hopping for NP [408] and then replacement of fragments in active compounds was reported more recently [409,410]. Other approaches, such as pseudo-NP [411,412], privileged scaffolds [413], and fragment libraries of NP, were also explored. The *pseudo-NP* libraries generated by Waldmann and co-workers [404] using diversity-oriented synthesis (DOS) such as ring-opening, ring-expansion, ring-contraction, or ring-rearrangement/fusion (Figure 11) occupy areas of chemical space not covered by NP and biology-oriented synthesis (BIOS) libraries. 

The chemical space of the *pseudo-NP* was compared by the authors with the NP in the ChEMBL database, the set of approved drugs by the DrugBank, and the BIOS libraries. It was observed that *pseudo-NP* has a narrower distribution that only covers a portion of the chemical space sparsely occupied by NP [404]. 

**Figure 12:**
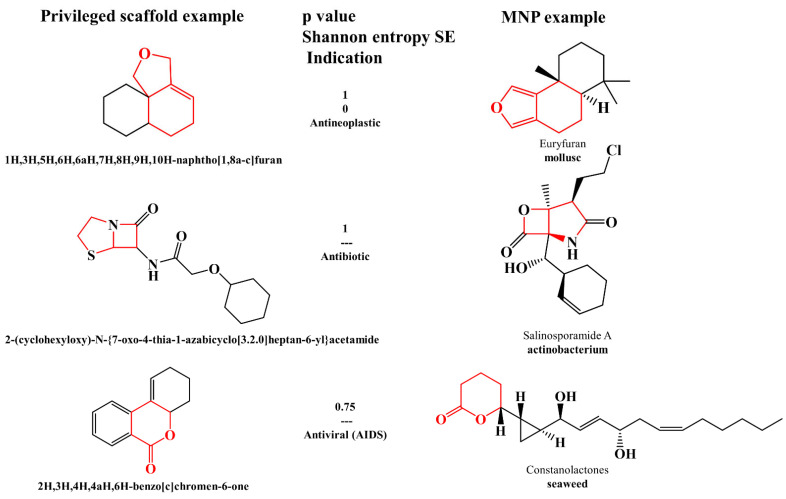
Examples of *privileged scaffolds* with the respective *p* value, SE value, and indication. These *privileged scaffolds* or similar were also found in MNP; representative examples are shown. Similar fragments are indicated in red [403,404,405,406].

Lai et al. reported a method using a deep learning approach to predict indications and identify *privileged scaffolds* of NP for drug design. Entropy-based information metrics were used to identify the *privileged scaffolds* for each indication, and a Privileged Scaffold Dataset (PSD) of NP was built. In Figure 12, some examples are shown [403,404,405,406].

**Figure 13:**
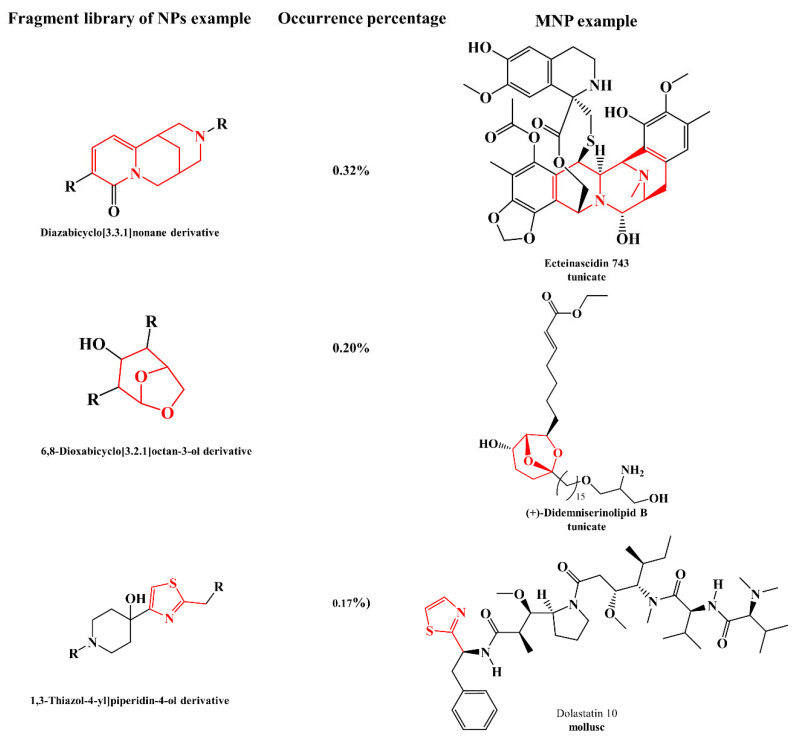
Examples of *fragment libraries* of NP with the respective occurrence percentages in COCONUT database. These *fragments* were also found in MNP; representative examples are shown. Similar fragments are indicated in red [10,13,406].

A large *fragment library* of NP with almost 206,000 fragments was recently reported by Chávez-Hernández et al. [406] from a drug-like subset of the COCONUT database using a Statistical-Based Database Fingerprint approach. COCONUT is available on Zenodo and comprises structures and some annotations for over 400,000 non-redundant NP [10,13]. The fragment library of NP was made freely available, and in Figure 13, some representative examples of this library are shown.

Recently, several works have been published using QSAR modeling to predict biological activities [409,410,414,415,416,417,418,419,420,421,422,423] or estimate absorption, distribution, metabolism, excretion, and toxicity (ADMET) properties [424,425] of NP, with special emphasis on MNP. The GDB4c database is a useful resource for similarity and pharmacophore searching based on known NP and is available for download at www.gdb.unibe.ch (accessed on 18 January 2023). An angle-based macrocycle conformational sampling method was explored by Wang et al. [411] using crystal structures of 37 polyketides with 9−22 rotatable bonds in the macrocyclic ring since macrocyclic polyketides are pharmacologically important NP. This method was able to reproduce the crystal structure of polyketides’ aglycone backbone within an RMSD of 0.50 Å for 31 out of 37 polyketides [411]. 

Two QSAR studies were developed from a seaweed metabolite database of marine algal secondary metabolites (http://www.swmd.co.in (accessed on 18 January 2023)) for predicting anticancer activity [412] against six different cancer cell lines (e.g., MCF-7, human breast adenocarcinoma; A431, human epithelial carcinoma; HeLa, human cervical adenocarcinoma; HT-29, human colon adenocarcinoma grade II; P388, murine leukemia; A549, human lung epithelial adenocarcinoma), antiamnestic and antidepressant activities against sigma receptors [413] using 157 [412] and 11,517 MNP [413], respectively. In the last study, 15 MNP were proposed as powerful sigma receptor ligands; four of them were already known in the literature for their antiproliferative and cytotoxic effects against A549 and HT29 cancer cell lines, which are two typical cancer cell lines characterized by sigma receptor overexpression [413]. In addition to anticancer activity, to discover new inhibitors against the human colon carcinoma HCT116 cell line, two QSAR studies using molecular and nuclear magnetic resonance (NMR) descriptors from 50 crude extracts, 55 fractions, and five pure compounds obtained from actinomycetes isolated from marine sediments collected off the Madeira Archipelago, were recently reported through exploration of ML techniques [426]. In this work, the two developed approaches (A, through molecular structures, and B, through NMR spectra) allowed the development of a complementary strategy to predict new anticancer MNP [426]. Approach B enabled the prioritization of the isolation, purification, and structural elucidation of crude extracts, fractions, and pure compounds. Therefore, pure compounds that were elucidated were subjected to model A, and the compounds predicted to be most active against the HCT116 cell line were evaluated experimentally [426]. Other QSAR studies reported anticancer activity models against protein targets such as heme oxygenase 1 (HO-1) [407] and p38α [408] from 62 molecules with HO-1 IC_50_ value ≤ 10 μM [407] and 45 brominated-based natural tyrosine synthetic derivatives [408] (a library that was synthetized based on the secondary metabolite isolated from the sponge *Iotrochota purpurea*, itampolin A), respectively. The virtual screening of new potentially HO-1 inhibitors of imidazole-based NP from three different databases, MNP (http://docking.umh.es/ (accessed on 18 January 2023)), ZINC NP, and Super Natural II, using the best QSAR model, was also reported by Floresta et al. [407].

Antifouling activity was QSAR modeled for the settlement of *Mytilus galloprovincialis* larvae [409,410]. Almeida et al. built two QSAR models using multilinear regression methods with 19 and 16 nature-inspired (thio)xanthone [409] and chalcone [410] derivatives, respectively, and also used in vitro antifouling activity assays for the settlement of *Mytilus galloprovincialis* larvae. Recently, Gaudencio and Pereira, 2022 performed a virtual screening antifouling campaign of 14,492 MNP from Encinar’s website and 14 MNP that are currently in the clinical pipeline. In the CADD structure-based approach, the 125 MNP that were selected by the QSAR approach were used in molecular docking experiments against the acetylcholinesterase enzyme. Sixteen MNP were proposed as the most promising marine drug-like leads as antifouling agents, e.g., macrocyclic lactams, macrocyclic alkaloids, indole, and pyridine derivatives [414].

QSAR modeling for the anticancer activity against HCT116 [426] and the antibacterial activity against methicillin-resistant *Staphylococcus aureus* (MRSA) infection [415] was also performed. The authors reported that the developed MRSA QSAR regression model, approach A, is the largest study ever performed with regard both to the number of compounds involved and to the number of structural families involved in the modeling of the antibacterial activity against MRSA [415,416,417,418]. The NMR QSAR classification model, approach B, was also extended to a high number of samples containing additional 45 pure compounds, and therefore the overall predictability accuracies were improved, [415] when compared with those obtained in their previous work [426]. 

The QSAR methodology was explored in the discovery of new antimalarial drugs of marine origin [419,420]. Aswathy et al. [419] studied 42 natural-based derivatives of thiaplakortone-A, which were found in the Australian marine sponge *Plakortis lita* and were active against chloroquine-sensitive and chloroquine-resistant *Plasmodium falciparum*. The authors reported several QSAR models, including both 2D and 3D QSAR, and the results were combined with simulated interactions with the *P. falciparum* calcium-dependent protein kinase 1 protein to design and screen new virtual molecules [419]. 

In another approach, quantitative relationships were established between thermodynamics/electronic properties calculated by DFT methods and the antimalarial activity of 14 sponge metabolites–bromopyrrole alkaloid derivatives [420]. The linear regression models were developed using molecular descriptors such as entropy, dipole moment, molecular polarizability, energy of the highest occupied molecular orbital (HOMO), softness, and electrophilicity index [420]. The HOMO also performed remarkably well in discriminating the overall biological activity of MNP and microbial NP [421].

The investigation of MNP as a key resource for the discovery of drugs to mitigate the COVID-19 pandemic is a developing field. Several CADD approaches were explored [422,423,427,428,429]. Gaudêncio and Pereira [424] reported a CADD ligand- and structure-based strategy for predicting marine SARS-CoV-2 main protease (M^pro^) inhibitors. A list of virtual screening hits comprising fifteen MNP was assented to by the authors on the basis of established limits, such as confidence value (3), probability of being active against SARS-CoV-2 in the best QSAR model, prediction of the affinity between the M^pro^ of the selected MNP through molecular docking, and ADMET predictions. Five MNP, benzo [f]pyrano [4,3-b]chromene, notoamide I, emindole SB beta-mannoside, and two bromoindole derivatives were proposed as the most promising marine drug-like leads as SARS-CoV-2 M^pro^ inhibitors [424].

**Figure 14:**
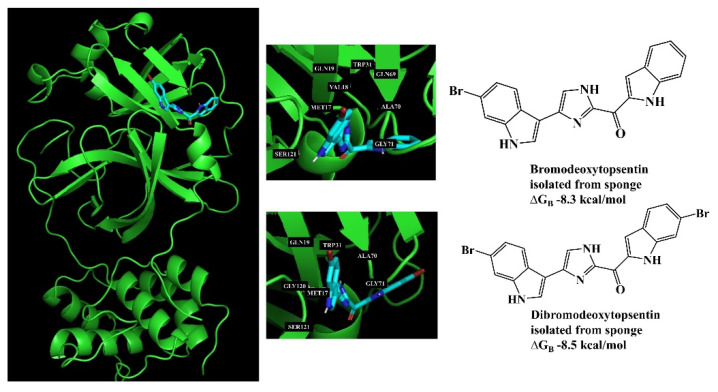
Interaction profiles of the best-docked poses for the two bromoindole hits in molecular docking to the M^pro^ enzyme (Protein Data Bank ID: 6LU7) [424].

In Figure 14, the interaction profiles of the best-docked poses for the two bromoindole lead-like SARS-CoV-2 M^pro^ inhibitors are shown [424].

Molecular docking has been the major structure-based methodology to predict affinities to macromolecular targets, interpret binding modes, and assist in the design of drug leads. Several recent publications illustrate the application of this method to MNP [424,425,430], and some representative examples are described herein.

**Figure 15:**
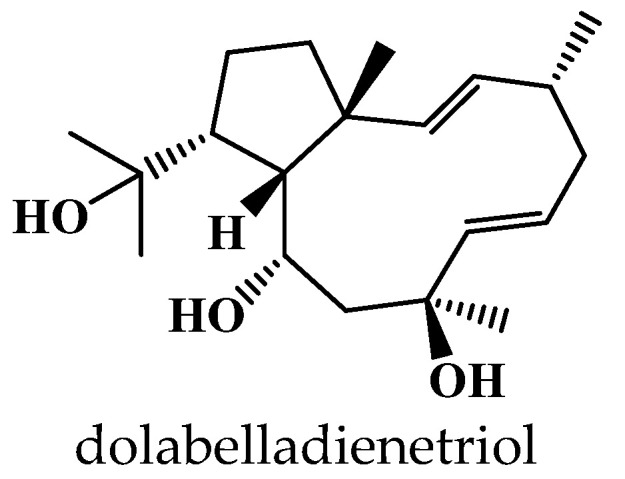
Chemical structure of dolabelladienetriol isolated from marine algae *Dictyota pfaffii*. Docking studies performed for cytotoxic NP isolated from Red Sea cucumber *Holothuria spinifera* revealed their binding interactions with the active site of the SET protein, an inhibitor of protein phosphatase 2A (PP2A), which could explain its cytotoxic activity [431].

Liu et al. [430] reported the design of a synthetic marine-based library comprising 19 tasiamide B (an acyclic peptide containing a statine-like unit and several amino acid residues) derivatives as inhibitors of BACE1, a potential therapeutic target for Alzheimer’s disease. The core structure and a free carboxylic acid group were identified as relevant for inhibitory activity by SAR analysis and docking simulation. vonRanke et al. [425] reported SAR, molecular docking, and molecular dynamic studies of ten diterpenes with anti-HIV activity that were previously isolated from marine algae and octocorals. In the SAR analysis, descriptors such as cLogP (octanol–water partition coefficient), PSA (polar surface area), LUMO (lowest unoccupied molecular orbital energy), and GAP_HOMO-LUMO_ (energy difference between the HOMO and LUMO) were identified, associating the anti-HIV activity of five diterpenes with possible action on the reverse transcriptase allosteric site. Further investigation by molecular docking identified that only dolabelladienetriol (Figure 15) interacted at the allosteric site. The high affinity of dolabelladienetriol for the allosteric site was confirmed by molecular dynamics analyses, which showed a hydrogen bond to Lys101 and a high hydrophobic interaction with the residues Leu100, Tyr318, Try188, Trp229, Val106, and Leu324. Based on molecular dynamics analysis, the authors suggested that dolabelladienetriol might interfere with the viral RNA binding to HIV-1 RT by inducing a conformational change of the enzyme.

## 9. Conclusions

It is of utmost importance to develop integrated, effective methods based on the use of a wide range of multidisciplinary technologies that enable researchers to prioritize natural resource (NR) samples, rapid dereplication, and evaluation of preferred cultivation and extraction conditions. Moving forward with rapid and efficient isolation, the discovery of novel specialized metabolites, and the 3D structural elucidation of the metabolite’s chemical scaffolds while minimizing the waste of resources on rediscovering known compounds, more and more chemists are using cutting-edge analytical and computational methods to organize and mine data from enormous data sets to accelerate the discovery of bioactive NP. MN was able to successfully organize extensive collections of MS/MS data as well as sample metadata in a format that was simple to understand for spectral similarity networks. The process of carrying out NP dereplication and metabolic profiling was significantly influenced by the online platform GNPS (Global Natural Products Social Molecular Networking), especially when combined with MN. 

The post-genomics era and the development of bioinformatic tools have also had a significant impact on NP research, speeding up the process of dereplication and structure elucidation of secondary metabolites.

Starting from the premise that the NR are integrated into their habitat, one should select the best approach and techniques to investigate the NR of interest based on an integrated approach to NP identification using the several technologies that are currently available. The unexpected diversity of the NR metabolome outweighs the complexity of the genome by a considerable margin. We are just starting to put together the necessary computational and experimental tools to understand the metabolome in comparable detail. We anticipate that in the future it will be possible to comprehend the precise connections between NR, their genome and metabolites, absolute structure elucidation, bioactivity, MoA, and immune response.

The diversity and pervasiveness of NR have been seen in new ways by modern technology, but these tools have primarily produced outlines that provide insufficient insight into organisms’ functions or community dynamics. Advancing knowledge about organisms’ functions or community dynamics could revolutionize our perception of the world and spark information and innovations in a variety of fields, including the environment, biotechnology, and health. Discovering the relationship between microbiome and NP structure would advance science towards increasing the NP chemical space and solving NR supply shortages for further biotechnological development such as pre-clinical and clinical trials or moving forward from proof of concept in industrial development.

## Figures and Tables

**Table 1:** Databases for MS/MS, GC-MS, IMS, and MALDI dereplication, MS/MS visualization and annotation tools, and MS/MS, GC-MS, IMS, and MALDI-MS processing informatic analysis tools.

Databases for MS/MS Dereplication				Databases for GC-MS, IMS, and MALDI Dereplication	
GNPS	GNPS/ MassIVE	Metabolitghts	MarinLit	GNPS/ MassIVE	MetaboLights
Metabolomics workbench	MassBank	ReSpect	NIST	MSHub/ GNPS	ProteomeXchange
MoNA	mzCloud	SPLASH	LipidXplorer	Metabolomics Workbench	PRIDE
Sumner/ Bruker	CASMI	PNNL Lipids	Sirenas/ Gates	**GC-MS, IMS, and MALDI-MS** **Processing Informatic Analysis Tools**	
EMBL	MCF	SistematX	NPBS	MSHub/ GNPS	
CMNPD	MIADB/ Beniddir	NPNPD	Antibase	SpeDE	
HMDB	MIADB	SistematX	DNP	AMDIS	
UNP	ChemSpider	Reaxys	SciFinder	RAMSY	
PubMed	Community-curated data	Users Libraries	-		
**MS/MS Visualization and Annotation Tools**					
PCA	PoPCAR	PLS-DA	MN		
MS2LDA	IIMN	NAP	DFMN-ISD		
FBMN	CLMN	BBMN	BMN		
MolNetEnhancer	MS2LDA-MOTIF	DEREPLICATOR	SIMILE		
MetaboAnalyst	MSDIAL	XCMS Online	HMDB		
Fragmentation Trees	GNPS Dashboard	Optimus and ‘ili	EMPress		
Qemistree	ChemProp	PPNet	-		
**MS/MS Processing** **Informatics Analysis Tools**					
GNPS Dashboard	MASST	GNPS	Mzmine.FBmn		
CAM	XCMS Online	HMDB	MSDIAL		
SPLASH	RMassBank	BinBase	MZmine		
Bioclipse	MSDK	SIRIUS 1 to 4	CSI:FingerID		
DEREPLICATOR	DEREPLICATOR+	NRPro	ReDU		
QIIME and QIIME 2	Qiita	CytoScape	Optimus and ‘ili		
MetaboAnalyst	MS/MS-Chooser	ChemProp	PPNet		
MeHaloCoA	SpeDE	ConCise	ClassyFire		
Bioclips	MSDK	XCMS	Qemistree		
EMPress	LLAMAS	NP Analyst	MetFrag		
MetFusion	MAGMa	MIDAS	FT-BLAST		
ISIS	FinderID	CFM-ID	MS-FINDER		
MetEX	MeTCirc	Spectrum_utils	COSMIC		
ZODIAC	CANOPUS	NPClassifier	IPO		
CAMERA	-	-	-		

**Table 2:** Databases for NMR dereplication and NMR processing informatics analysis tools.

Databases for NMR Dereplication	
Antibase	MarinLit
NP-MRD	NP Atlas
MIBiG	StreptomeDB 3.0
PNMRNP	COCONUT
UNP	KnapsackSearch
NPBS	CMNPD
**NMR Processing Informatic Analysis Tools**	
HiFSA	MixONat
XGBoost classifier	2D barcodes
NMRfilter	COLMAR
SMART	SMART 2.0
SMART- Miner	MatchNat
DEREP-NP	MADByTE
RESTful	NP Classifier
ClassyFire	CyanoMetDB

## Data Availability

Not applicable.
